# Genome Duplication Increases Meiotic Recombination Frequency: A *Saccharomyces cerevisiae* Model

**DOI:** 10.1093/molbev/msaa219

**Published:** 2020-09-08

**Authors:** Ou Fang, Lin Wang, Yuxin Zhang, Jixuan Yang, Qin Tao, Fengjun Zhang, Zewei Luo

**Affiliations:** 1 Laboratory of Population and Quantitative Genetics, Institute of Biostatistics, Fudan University, Shanghai, China; 2 Qinghai Academy of Agriculture and Forestry Sciences, Xining, Qinghai, China; 3 School of Biosciences, University of Birmingham, Birmingham, United Kingdom

**Keywords:** meiotic recombination frequency, genome duplication, tetrasomic linkage analysis, *S. cerevisiae*

## Abstract

Genetic recombination characterized by reciprocal exchange of genes on paired homologous chromosomes is the most prominent event in meiosis of almost all sexually reproductive organisms. It contributes to genome stability by ensuring the balanced segregation of paired homologs in meiosis, and it is also the major driving factor in generating genetic variation for natural and artificial selection. Meiotic recombination is subjected to the control of a highly stringent and complex regulating process and meiotic recombination frequency (MRF) may be affected by biological and abiotic factors such as sex, gene density, nucleotide content, and chemical/temperature treatments, having motivated tremendous researches for artificially manipulating MRF. Whether genome polyploidization would lead to a significant change in MRF has attracted both historical and recent research interests; however, tackling this fundamental question is methodologically challenging due to the lack of appropriate methods for tetrasomic genetic analysis, thus has led to controversial conclusions in the literature. This article presents a comprehensive and rigorous survey of genome duplication-mediated change in MRF using *Saccharomyces cerevisiae* as a eukaryotic model. It demonstrates that genome duplication can lead to consistently significant increase in MRF and rate of crossovers across all 16 chromosomes of *S. cerevisiae*, including both cold and hot spots of MRF. This ploidy-driven change in MRF is associated with weakened recombination interference, enhanced double-strand break density, and loosened chromatin histone occupation. The study illuminates a significant evolutionary feature of genome duplication and opens an opportunity to accelerate response to artificial and natural selection through polyploidization.

## Introduction

In meiosis of all sexually reproductive eukaryotic species, recombination between homologous chromosomes generates reciprocal exchanges of genes on the chromosomes through chromosomal crossing over. Crossovers (COs) are essential for ensuring physical connections and balanced segregation between the homologous chromosomes (Jones and Franklin 2006). Reciprocal exchange of parental genetic material allows reshuffling of genes on these chromosomes and thus creates new allelic combinations of them in offspring individuals for natural and/or artificial selection. It has been well documented that meiotic recombination is a strictly programmed and controlled process, characterized by DNA double-strand breaks (DSB) catalyzed by the topo-isomerase-related enzyme Spo11 and repairing of the breaks involving with a group of evolutionarily highly conserved proteins (Osman et al. 2011). It is well established that initiation and distribution of recombination along chromosomes are not random, and meiotic recombination frequency (MRF) may be affected by several biological and genomic factors such as sex (Lenormand and Dutheil 2005), density of genes or transposable elements (Shifman et al. 2006), GC nucleotide content (DeRose-Wilson and Gaut 2007), and genome sequence heterozygosity (Ziolkowski et al. 2015).

Whether polyploidization of a eukaryotic genome alters the MRF has attracted both historical and recent research interests. Oram (1959) was probably the pioneer in investigating this fundamental question by comparing MRF of two chromosomal regions in diploid and tetraploid maize segregation populations and concluded that MRF in one of the two marker intervals was significantly higher in tetraploids than that in diploids, but the difference is not significant in the other chromosomal interval. Using data collected on three linked dominant/recessive markers from diploid and autotetraploid maize backcross populations, Welch (1962) observed an increased rate of COs in diploids in one of the two linked chromosomal intervals but no difference in the CO rate in the other, and emphasized the weakness of his data analysis for unavailability of an appropriate method for analyzing the autotetraploid data. Sved (1964) presented a theoretical prediction that polyploidization through genome duplication may increase MRF. More recently, Pecinka et al. (2011) focused on recombination frequency between a single pair of seed expressing fluorescent markers segregating in large diploid and tetraploid populations of *Arabidopsis* and concluded that recombination frequency between the fluorescent marker loci was markedly higher in the tetraploids than in the diploids. Recognized inappropriate method of statistical analysis with the tetraploid marker data, we reanalyzed their data set and inferred a significant increase in the recombination frequency in the tetraploids than in the diploids (Wang and Luo 2012). This article reports a comprehensive study on ploidy-driven change in MRF by using *Saccharomyces cerevisiae* as an experimental model and by developing appropriate statistical methods for modeling and analyzing the autotetraploid experimental data sets.

## Results

### Creation and Transformation of Fluorescent Marker Cassettes

**Fig. 1.**
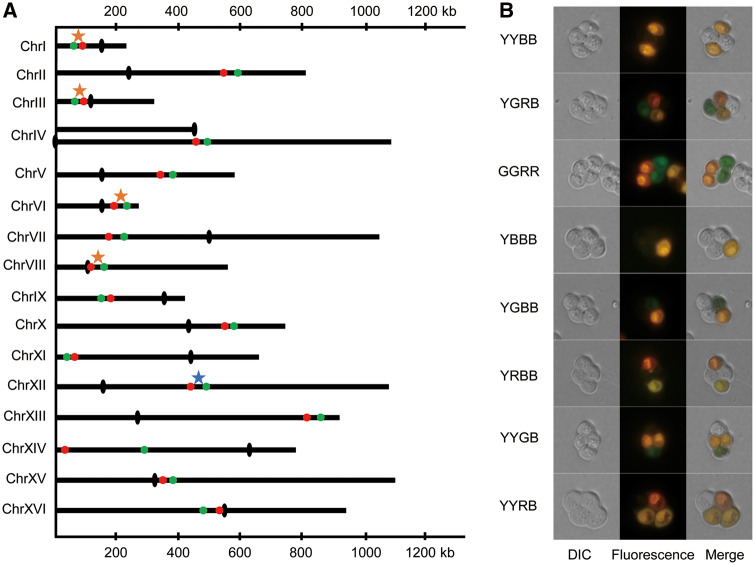
(*A*) Distribution of fluorescent red and green markers on each of 16 yeast chromosomes with black dots indicating the centromeres, the red and blue stars being the previously defined hot and cold recombination spots. (*B*) Phenotype of spores carrying different alleles at the fluorescent markers. The black olives indicate the centromeres on the yeast chromosomes.

Firstly, we modified the lithium acetate method (Lorenz et al. 1995) to transform the two fluorescent cassettes, *GFP* and *RFP* (supplementary fig. 1*A*, Supplementary Material online), which were driven by the promoter *TEF2* and *TDH1*, onto the same chromosome at predesigned locations in the haploid yeast strain S288c. We then developed a feasible but reliable experimental approach to convert two haploid yeast strains, S288c and SK1, respectively, into diploid and then duplicated the diploids into autotetraploid strains as described in supplementary fig. 1*B* and supplementary method, Supplementary Material online. Ploidy levels of the created diploid and autotetraploid strains were confirmed in terms of measure of the genomic DNA content by use of the fluorescence-activated cell sorting (supplementary fig. 1*C*, Supplementary Material online). We repeated transformation of the cassette bearing the two fluorescent markers into each of the 16 chromosomes at a predesigned location as illustrated in figure 1*A*. Precise locations of these fluorescent marker genes are detailed in supplementary table 1, Supplementary Material online, for each of the 16 yeast chromosomes. Design of the marker locations was mainly arbitrary for a fairly even representation of the marker locations in the 16 yeast chromosomes except that some of these marker genes have been deliberately designed to locate within previously identified recombination hot (red stars) or cold (blue stars) spot regions (Gerton et al. 2000) as highlighted in figure 1*A* and supplementary table 1, Supplementary Material online.

For each of the fluorescent marker cassettes, we created two F_2_ segregating populations from crossing the two parental strains, s288cand SK1, in diploid and autotetraploid, respectively, and scored a varying number (603–2,129) of tetrads from these segregating populations for their phenotype of the two fluorescent markers (supplementary table 2, Supplementary Material online). Each spore in the 26,281 tetrads scored was phenotyped as either black (B), green (G), red (R), or yellow (Y), corresponding to the spores that carry none, only green allele, only red allele, or both of the fluorescent marker alleles (fig. 1*B*).

### Meiotic Recombination Frequencies in the Diploid and Autotetraploid Segregating Populations

The tetrad data are fully informative in regard to the underlying genotype and recombination events during meiosis of the diploid parents, and thus calculation of recombination frequency between the fluorescent markers is straightforward in the diploid segregating populations. However, the same analysis in the autotetraploid segregating populations is far more complicated, primarily due to the complexities in gene segregation and recombination under tetrasomic inheritance, reflecting in several main aspects. Firstly, in autopolyploids, homologous chromosomes in meiosis may undergo quadrivalent pairing, resulting in the well-known phenomenon of double reduction, that is, sister chromatids enter into the same gamete (Mather 1936) after the meiosis, leading to systematic allelic segregation distortion in comparison to disomic gene segregation and recombination. Secondly, multiple alleles at individual loci of polyploids cause a substantially wider spectrum of genotypic segregation at the loci. These make tetrasomic linkage analysis a historically challenging and unsolved question (Bailey 1961) since the pioneer geneticists like Fisher (1947), Haldane (1930), and Mather (1936). We have developed a general statistical framework for tetrasomic linkage analysis, which has taken a full account of the key features of tetrasomic inheritance (Luo et al. 2004). We modified that general framework to specifically model and analyze the tetrad data of the autotetraploid segregating populations in the present study as detailed in Statistical Method 1.

**Fig. 2.**
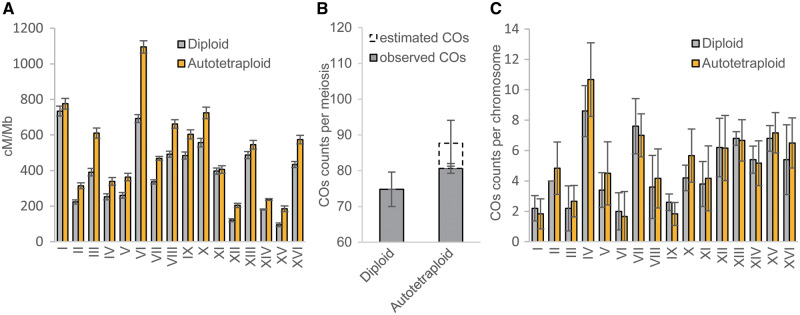
(*A*) The estimated linkage map distance (cM) normalized by the corresponding physical map distance (Mb) and (*B*) means of the observed or/and predicted number of COs per meiosis of diploids and autotetraploids. (*C*) The observed number of COs per meiosis across 16 chromosomes (I–XVI) from *n *=* *5 diploid tetrads (HS) and *n *=* *6 autotetraploid tetrads (hsss or hhhs) yeast *Saccharomyces cerevisiae*.

Table 1lists the maximum likelihood estimates of recombination frequencies ($\hat{r}$) and their sampling standard deviations (SD) between the fluorescent markers across the 16 yeast chromosomes in autotetraploid and diploid yeast genomes of *S. cerevisiae*. It shows that MRF in the autotetraploid genome is consistently highly significantly increased across all the 16 yeast chromosomal regions under investigation when compared with that in the diploid genome. We have converted these estimates of MRF into mapping distances in cM by multiplying the MRF estimates by 100 and illustrated the estimated mapping distances of the marker regions normalized by the corresponding physical distance in Mb in figure 2*A*. Means of cM/Mb values are calculated to be 383.5 and 506.7 for the diploid and tetraploid yeasts, respectively. It is noted that the significant increase in MRF in the autotetraploids was also observed in both previously characterized hot or cold recombination spots (supplementary table 1, Supplementary Material online). Listed also in table 1 are the maximum likelihood estimates of the coefficient of double reduction at the linked fluorescent marker loci ($\hat{\alpha }$and $\hat{\beta }$) and the corresponding standard deviations. Moreover, estimates of the coefficient of double reduction are statistically significant for the markers on chromosomes 4, 7, 13, 14 (table 1), revealing a clear signal of the quadrivalent chromosomal pairing in the marked chromosomal regions during meiosis of the autotetraploid yeast cells, suggesting significant double reduction event and tetrasomic inheritance of maker alleles in the autotetraploid yeast strains. Additionally, we tested for significance in allelic deviation from neutral segregation at the marker loci and none of the tests was statistically significant (*P* ≥ 0.1750), excluding the possibility of selection on the fluorescent markers and the influence of selection on assessing MRF.

**Table 1.** Maximum Likelihood Estimates of Recombination Frequencies ($\hat{r}$) and the Sampling Standard Deviations (SD) between the Fluorescent Markers Across 16 Yeast Chromosomes in Autotetraploid and Diploid Yeast Genomes as well as the Maximum Likelihood Estimates of the Coefficient of Double Reduction at the Linked Fluorescent Loci ($\hat{\alpha }$and $\hat{\beta }$) and the Corresponding Standard Deviation.

Chromosomes	Autotetraploids			Diploids
	$\hat{\alpha }\pm \text{SD}$	$\hat{\beta }\pm \text{SD}$	$\hat{r}\pm \text{SD}$	$\hat{r}\pm \text{SD}$
1	0.0305 ± 0.0336	0.0361 ± 0.0335	0.2245 ± 0.0086	0.2126 ± 0.0081
2	0.0466 ± 0.0394	0.0435 ± 0.0393	0.1640 ± 0.0088	0.1165 ± 0.0065
3	0.0060 ± 0.0347	0.0229 ± 0.0347	0.1617 ± 0.0076	0.1035 ± 0.0056
4*	0.0659 ± 0.0382	0.0629 ± 0.0382	0.1071 ± 0.0069	0.0797 ± 0.0054
5	0.0431 ± 0.0392	0.0446 ± 0.0392	0.1404 ± 0.0081	0.1004 ± 0.0061
6	0.0147 ± 0.0383	0.0368 ± 0.0383	0.4392 ± 0.0139	0.2776 ± 0.0088
7-1**	0.0618 ± 0.0233	0.0678 ± 0.0234	0.2322 ± 0.0062	0.1599 ± 0.0062
7-2**	0.0629 ± 0.0216	0.0672 ± 0.0217	0.2445 ± 0.0059	0.1755 ± 0.0063
8	0.0000 ± 0.0383	0.0063 ± 0.0398	0.3460 ± 0.0128	0.2571 ± 0.0087
9	0.0252 ± 0.0385	0.0312 ± 0.0385	0.2389 ± 0.0103	0.1918 ± 0.0080
10	0.0338 ± 0.0392	0.0510 ± 0.0392	0.2295 ± 0.0103	0.1765 ± 0.0076
11	0.0404 ± 0.0387	0.0510 ± 0.0387	0.1682 ± 0.0087	0.1645 ± 0.0074
12	0.0495 ± 0.0361	0.0326 ± 0.0361	0.1085 ± 0.0065	0.0642 ± 0.0046
13*	0.0786 ± 0.0377	0.0814 ± 0.0378	0.2110 ± 0.0095	0.1884 ± 0.0078
14**	0.0637 ± 0.0308	0.0751 ± 0.0308	0.6143 ± 0.0133	0.4681 ± 0.0083
15	0.0000 ± 0.0403	0.0098 ± 0.0403	0.0650 ± 0.0056	0.0332 ± 0.0036
16	0.0000 ± 0.0384	0.0217 ± 0.0394	0.2644 ± 0.0110	0.1999 ± 0.0080

Note.—

* (*P* < 0.05) and

** (*P* < 0.01) indicate the creditability levels of significance of estimates of the coefficients of double reduction at the fluorescent markers.

### Rate of COs in Diploid and Autotetraploid Yeast Genomes

We further characterized the genome-wide distribution of COs generated in heterozygous diploid and autotetraploid yeast strains. We first created a heterozygous diploid strain s288c/SK1 (or hs in abbreviation) from the haploid strains, s288c and SK1, which differ at least at 63,000 single nucleotide polymorphism (SNP) sites (1 SNP per 190 bp or per 0.06 cM) (Gerton et al. 2000). We designed autotetraploid strains with genome constructs of s288c/SK1/SK1/SK1 (or hsss) and s288c/s288c/s288c/SK1 (or hhhs) (Materials and Methods). These designed constructs allow CO detection to be focused on the same single chromosome in both diploid and autotetraploid genomes, and thus enable a direct comparison of rate of CO involved with the specific single chromosome between diploid and tetraploid genomes. We randomly collected and microdissected five, three, and three tetrads generated from the diploid (hs), autotetraploid (hsss), and autotetraploid (hhhs) strains, respectively, using a dissection microscope (SINGER MSM400, United Kingdom). These tetrads from heterozygous diploid and autotetraploid strains were sequenced using Illumina’s Hiseq 2000 sequencer with a design of 2 × 100 bp paired-end reads.

From the tetrad sequence data sets, we firstly identified the sequence variant marker sites for the CO analysis, on which the marker alleles show a 2:2 allele configuration in diploid tetrads (hs), a 6:2 or 2:6 configuration in tetraploid tetrads (hhhs) or (hsss), respectively, and thus selected an average of 47,700 markers from the diploid tetrads and an average of 51,969 markers from the tetraploid tetrads for assaying rate of COs in these tetrad spores. Use of these selected sequence-based markers may effectively avoid influence of sequencing errors, errors from data processing such as nucleotide calling, sequence reads mapping, and the compounding meiotic events involving with gene conversion or structural events due to genome instability. A CO was identified as a reciprocal exchange occurring between chromatids marked by the selected marker sites (supplementary fig. 2, Supplementary Material online). We were able to observe COs directly from the diploid tetrad sequence data and from part of tetraploid tetrad sequence data when linkage phase at the linked marker loci can be directly inferred. The COs so derived are referred to as observed COs.

However, it is not feasible to call COs directly from the autotetraploid tetrad sequence data because any tetrad spore is a diploid and linkage phase may be unknown for any spore with a double heterozygote genotype at the flanking sequence markers. We proposed here a statistical method for predicting the number of COs per chromatid of an autotetraploid from the tetrad sequence data as detailed in Statistical Method 2. Using the method, we calculated the expected number of COs for each chromosome in the autotetraploid genomes. The COs so derived are referred to as estimated COs. To further remove those observed COs which may be vulnerable to the sequence errors and sequence variants aforementioned, we removed those adjacent observed COs if they were separated by <10 kb (Malkova et al. 2004).

Figure 2*B* shows the mean number of COs observed from diploid yeast tetrads and observed plus estimated COs from tetraploid tetrads. A *t*-test shows that the mean number of observed COs from the tetraploid tetrads was significantly higher than that from the diploid tetrads (*P* < 0.05). The difference would be significant at a much higher statistical confidence if the comparison is made after including the estimated COs of the tetraploid tetrads. It is noted that difference in mean observed number of the observed COs (i.e., hhhs and hsss) was not significant between the two types of tetraploids (*t*-test with *P* = 0.8275). Figure 2*C* (and also supplementary table 6, Supplementary Material online) shows means of the observed COs for each of 16 chromosomes of the diploid and tetraploid yeast tetrads. A pairwise *t*-test shows significant difference in the mean number of observed COs across the 16 chromosomes between diploid and tetraploid yeasts (*P* < 0.05), and the corresponding test was not significant when comparison was between the two tetraploid types (*P* = 0.85).

### Comparison in Recombination Interference between Diploid and Autotetraploid Genomes

It has been well established that recombination does not independently occur along chromosome arms, and any recombination at a site may usually prevent others at any nearby sites, the phenomenon is so-called recombination interference (RI) (Keeney et al. 1997). RI may be attributed to two types of interference, the chromatid interference, where different pairs between nonsister chromatids are not equally likely to be involved in formation of COs, and the position or chiasmata interference, where occurrence of one CO event at a position along the chromosomal bundle affects chance of an additional CO to occur in a nearby region (Mieczkowski et al. 2007). We focus here on the latter, and test for a hypothesis that increased MRF in the autotetraploid yeast when compared with that in the diploid genome is in association with weakened RI.

To test the hypothesis, we created diploid and autotetraploid yeast strains which carried three antibiotic markers (*NAT*, *HGY*, and *G418*) in simplex dose on the same arm of the yeast chromosomes III, VI, and VIII, and details of these antibiotic markers are listed together with their chromosome locations in supplementary table 2, Supplementary Material online. These diploid and autotetraploid strains, respectively, generated the corresponding segregant (gamete) populations. Segregants of the diploid strain were haploids but segregants of the autotetraploid are diploids. The antibiotic markers are indeed dominant “present” and “absent” markers, and the number of diploid and autotetraploid segregants is listed in supplementary table 3, Supplementary Material online, together with the phenotype of the markers on each of the three yeast chromosomes.

Statistically, RI is defined as the coincidence coefficient (CC) in several different forms, which measures degree of independence of recombination events along two adjacent marker intervals flanked by marker loci A, B, and C. Let $r_{\text{AB}\cap \text{BC}}$ be frequency of recombination simultaneously occurring in the two adjacent marker intervals AB and BC. Let $r_{\text{AB}|\text{BC}}$ (or $r_{\text{AB}|\bar{\text{BC}}}$) be frequency of recombination between marker loci A and B with (or without) recombination in the interval flanked by markers B and C. The CC can then be defined as $c_{\text{AB}-\text{BC}}=r_{\text{AB}\cap \text{BC}}/(r_{\text{AB}}\times r_{\text{BC}})$, $c_{\text{AB}/\text{BC}}=r_{\text{AB}/\text{BC}}/r_{\text{AB}|\bar{\text{BC}}}$, and $c_{\text{BC}/\text{AB}}=r_{\text{BC}/\text{AB}}/r_{\text{BC}|\bar{\text{AB}}}$. In absence of RI, $c_{\text{AB}-\text{BC}}=c_{\text{AB}|\text{BC}}/c_{\text{BC}|\text{AB}}=1$ and significant deviation of the CC from the expected value of 1.0 imply significance of RI. In populations of the diploid segregants that are haploids, recombination events across the antibiotic marker intervals are directly countable, and the CCs can thus be calculated directly from the marker data and the estimates are listed in table 2. To test for significance of the CC estimates, we proposed here a simulation based method to generate an empirical significant thresholds for the CC estimates’ deviation from the null hypothetical value, and assessed significance of deviation of the CC estimates from their expected value of 1.0 under the null hypothesis. The simulation mimics gametogenesis of any given diploid genotypes at any number of loci assuming independent recombination among different chromosomal regions as detailed elsewhere (Luo et al. 2004), and the simulation parameters were extracted from the real data sets. It can be seen from table 2 that RI in the diploid data was detected significant in the marked region on chromosome III, highly significant on chromosome VI but not significant on chromosome VIII. It is further shown that the CC estimates and their significance were consistent each other, suggesting the equivalence and reliability of the CC estimates from the antibiotic marker data.

**Table 2.** The Maximum Likelihood Estimates of the Coincident Coefficients (${\hat{c}}_{\text{AB}-\text{BC}}$,${\hat{c}}_{\text{AB}/\text{BC}}$, and ${\hat{c}}_{\text{BC}/\text{AB}}$) from Diploid and Autotetraploid Yeast Chromosomes III, VI, and VIII and the Maximum Likelihood Estimates of the Coefficient of Double Reduction ($\hat{\alpha }$) and Its Sampling Standard Deviation ($s_{\hat{\alpha }}$) at Each of Antibiotic Markers (*NAT*, *HGY*, and *G418*) on Each of the Autotetraploid Yeast Chromosomes (III, VI, and VIII).

Diploids	Chromosome III			Chromosome VI			Chromosome VIII		
${\hat{c}}_{\text{AB}-\text{BC}}$	0.8214*			0.5990***			0.9436		
${\hat{c}}_{\text{AB}/\text{BC}}$	0.7695*			0.5727***			0.9319		
${\hat{c}}_{\text{BC}/\text{AB}}$	0.7978*			0.5322***			0.9241		
Autotetraploids	*NAT*	*HGY*	*G418*	*NAT*	*HGY*	*G418*	*NAT*	*HGY*	*G418*
$\hat{\alpha }$	0.0281	0.0506	0.0787	0.0201	0.0718	0.0316	0.0000	0.0315	0.0287
$s_{\hat{\alpha }}$	0.0530	0.0531	0.0536	0.0536	0.0536	0.0539	0.0535	0.0535	0.0535
${\hat{c}}_{\text{AB}-\text{BC}}$	1.0426			1.0376			0.8778		
${\hat{c}}_{\text{AB}/\text{BC}}$	1.0593			1.0442			0.8488		
${\hat{c}}_{\text{BC}/\text{AB}}$	1.0492			1.0557			0.8414		

Note.—

* *P *<* *0.05,

*** *P *<* *0.001.

The *P* values were calculated from 1,000 permutation simulations.

It is much more sophisticated to model and analyze the marker data for predicting RI from the autotetraploid segregants which are diploids because of the possible tetrasomic inheritance at the antibiotic markers. We first tested for significance of double reduction at each of the marker loci on each of the chromosomes under question. For instance, let *α* be the coefficient of double reduction at a marker locus A. Given an autotetraploid genotype ABBB, the autotetraploid generates two types of gametes AB and BB with probabilities (2−α)/4 and (2+α)/4, respectively, following the principle we previously developed (Luo et al. 2004). In an sample with $n_{1}$ of AB and $n_{2}$ of BB gametes, the maximum likelihood estimate of α is given by $\hat{\alpha }=2(n_{2}-n_{1})/(n_{1}+n_{2})$, and sampling variance of the estimate can be calculated as $s_{\hat{\alpha }}^{2}={\left(4-\alpha^{2}\right)}^{2}/[{\left(2+\alpha \right)}^{2}n_{1}+{\left(2-\alpha \right)}^{2}n_{2}]$. The maximum likelihood estimates of the coefficient of double reduction at the marker loci are listed in lower panel of table 2 together with the corresponding standard deviations. It shows that double reduction is not significant on every marker loci, suggesting bivalent pairing of homologous chromosomes at the loci under question during meiosis.

Based on the quadrivalent chromosomal pairing and simplex marker alleles linked on the same chromosome, we calculated the CC from the tetraploid segregant marker data. Again, we implemented a simulation-based approach to evaluate significance of the CC estimates’ deviation from their expected value of 1.0 under the null hypothesis. The simulation mimics gametogenesis of any autotetraploid genotype at any number of loci under either bivalent pairing or quadrivalent pairing of homologous chromosomes as previously described (Luo et al. 2004). Specifically, the simulated parental strain had a genotype ABC/abc/abc/abc where the capital letters correspond to the three antibiotic marker alleles, and other simulation parameters such as the number of segregants and recombination frequencies between the marker loci were directly extracted from the real data sets. The simulation was repeated 1,000 times and recombination was simulated independently among the two marker intervals. Table 2 shows that none of the marked chromosomal regions in the tetraploid yeast was detected to show significant RI. Comparison of the RI estimates for the same marked chromosomal regions in the same genome but at different ploidy levels indicates that RI in the tetraploid yeast genome was significantly weakened when compared with that in the corresponding diploid genome.

### DSB and Histone Occupation in Diploid and Autotetraploid Genomes

Crossing over between paired homologous chromosomes in meiosis is initiated by developmentally programmed DNA DSB, which are catalyzed by the topoisomerase-like protein Spo11 (Keeney et al. 1997). Change in DSB frequency will thereby affect frequency of COs and, in turn, recombination frequency (Mieczkowski et al. 2007). In the yeast strains with RAD50S mutated, the epitope-tagged Spo11 remains bound to the sheared DNA even after completion of DSB. This allows the DNA fragments surrounding the DSB sites to be enriched through immunoprecipitation of the Spo11–DNA complex (Alani et al. 1990; Gerton et al. 2000; Prieler et al. 2005). On basis of the principle, we compared the density and frequency of DSB in the diploid and autotetraploid yeast genomes. Immunoprecipitated Spo11-oligos from diploids and autotetraploids were deeply sequenced with two biological replicates for each of the two yeast strains. We obtained more than 1.5 million sequence reads of 50 bp length for per sample. More than 80% of the sequence reads of the sequenced samples (two biological replicates for diploid and tetraploid cells) were uniquely mapped to the genome of the yeast strain SK1, suggesting a good quality of the sequencing data. In parallel, genomic DNA from the strain SK1 was also sequenced as input control with the same sequence depth in order to achieve a comparable number of uniquely mapped sequence reads to that of the corresponding ChIP-seq experiment. We split the yeast genome into bins of 1 kb and calculated RPKM (reads per kilobases per million mapped reads) from the ChIP-seq and input control sequence data of the diploid and autotetraploid samples.

**Fig. 3.**
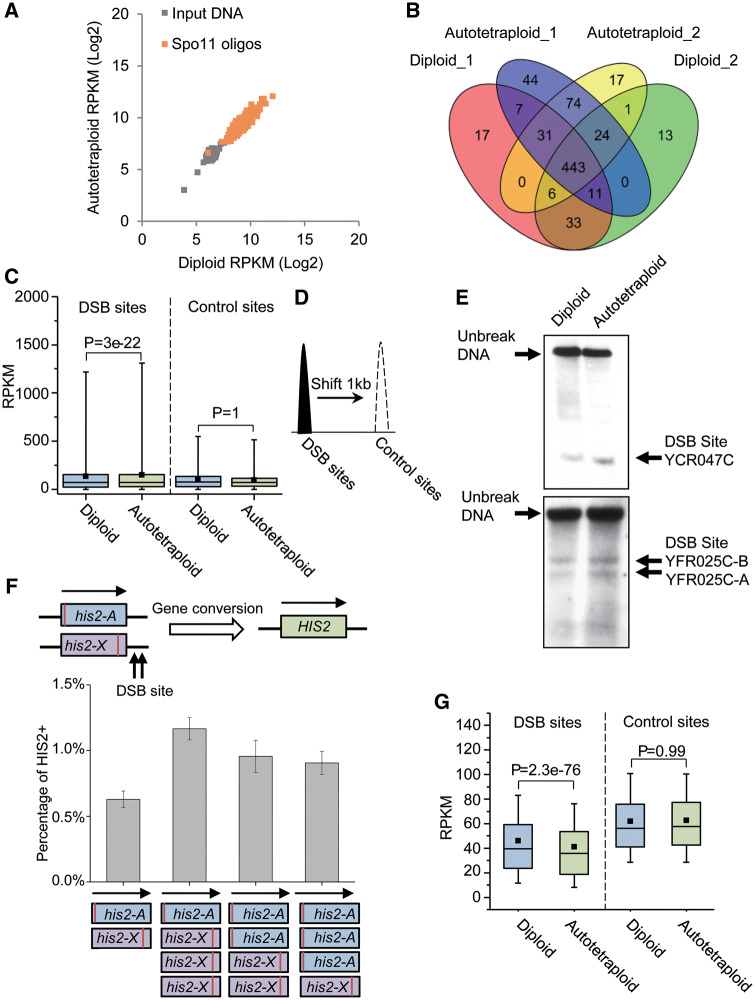
ChIP-seq assays of DSB and histone occupation in diploid and autotetraploid yeast strains. (*A*) Scatterplot of log_2_-transformed RPKM values of input DNA (gray) and spo11-oligos at the 443 DSB sites common to both diploid and autotetraploid cells. (*B*) The number of DSB sites identified from two biological replicates of independently cultured diploid and autotetraploid cells. (*C*) Illustration of ChIP-seq assay at the 3,600 DSB sites and control sites of 1 kb downstream of the corresponding DSB sites. (*D*) Boxplots of RPKM from ChiP-seq assay at the 3,600 DSB sites and the control sites in diploid and tetraploid cells. (*E*) Southern blotting assay of DSB density at three identified sites in genomes of diploid and tetraploid yeasts. (*F*) Rerun to growth assay of DSB at site *HIS2* (or YFR025C). (*G*) Boxplots of RPKM of MNase-resistant mononucleosome DNA at the 3,600 DSB sites and the control sites in diploid and tetraploid cells.

The calculated RPKM per bin from the diploid ChIP-seq data was highly significantly positively correlated with that from the autotetraploid ChIP-seq data (*r* = 0.92, *P* = 0), suggesting a high degree of consistency in distribution of DSB across the genome between the yeast strains at two different ploidy levels (fig. 3*A*). Among the enriched regions with an enrichment fold change of ≥3 observed in the diploid and autotetraploid samples, there were 443 shared by the two samples (fig. 3*B*), which include previously identified DSB hotspots, such as HIS4, HIS2, ARG4, and CYS3 (Gerton et al. 2000).

By sequencing Spo11-bound oligos, Pan et al. (2011) identified 3,600 DSB hotspots in diploid budding yeast, which included all the 443 enriched bins we detected in the present study. We compared the Spo11-oligos between the diploid and tetraploid at the 443 bins and all the 3,600 hotspots through a paired *t*-test. The test showed the autotetraploid fold enrichment was consistently significantly higher than that of the diploid at the 3,600 DSB sites (*P* = 3e-22, fig. 3*C*). In contrast, no difference was observed in the fold enrichment at the control sites (1 kb downstream of the DSB sites, fig. 3*D*) between the diploid and autotetraploid samples (*P* = 1.0, fig. 3*C*).

To further validate the ChIP-seq analysis, we compared the DSB density of diploid and autotetraploid yeasts at several previously identified sites, one at YCR047C and two within 3′ region of YFR25C (Petes 2001), through the standard Southern blotting assay in which the target DNA was detected by the probes labeled with digoxin-11-dUTP. The Southern blotting assay shows that DSB density at these sites was markedly higher in the tetraploid than in the diploid (fig. 3*E*), agreeing with that revealed by the ChIP-seq data. Moreover, the higher DSB frequency at 3′ region of YFR025C was also confirmed by an additional return-to-growth assay (fig. 3*F*). Additionally, we conducted the return-to-growth assay using genetically modified diploid and autotetraploid strains. The diploid strain carried two nonsense mutants, his2-A (blue box) and his2-X (purple box) of HIS2, whereas the corresponding autotetraploid carried two combinations of the mutant alleles on a chromosome (fig. 3*F*). The mutant carriers were auxotrophy but would recover back to be normal when a functionally normal HIS2+ was created from DSB and the following gene conversion between the two mutant genes on different chromosomes. Thus, proportion of the yeast cells carrying the mutant alleles, which could grow on the medium lacking of histidine (i.e., HIS2+ carrying cells), reflects the density of DSB surrounding the gene conversion. The right panel of figure 3*F* shows the percentage of the HIS2+ carrying cells with either a diploid or autotetraploid genome and reveals that the autotetraploid yeast had significantly higher density of DSB than the diploid cells. This provides further evidence supporting the autotetraploid genome has a denser DSB than the diploid and agrees well with the above ChIP-seq and Southern blotting assays.

Majority of DSB sites or CO hotspots are nuclease-hypersensitive and share a common open chromatin structure without nucleosome occupation, which are recognized to be necessary for Spo11 to access the DNA substrate and break DNA double strands (Petes 2001). The 3,600 DSB hotspots aforementioned were also observed to be markedly less occupied by nucleosomes (Prieler et al. 2005). We examined the histone occupation landscape in both diploid and autotetraploid genomes by sequencing DNA at micrococcal nuclease (MNase)-resistant mononucleosomes. The RPKM was calculated for per chromosome from the DNA sequence data and compared between the diploids and autotetraploids. The analysis shows that the RPKM of the autotetraploids is significantly decreased when compared with that of the diploids at all the 3,600 DSB sites (*P* = 2.3e-76, fig. 3*G*), indicating that the autotetraploid chromosomes were less wrapped by nucleosomes than the diploid chromosomes. This agrees well with the above ChIP-based DSB assay and further supports the ploidy-driven increase in MRF in budding yeast.

RPKM was proposed to detect differential gene expression between two samples from RNA sequence data when total amount of expression is comparable between samples under comparison (Evans et al. 2018). We implemented the normalization method to compare level of Spo11 bound and MNase-resistant oligos at the DSB sites between diploid and tetraploid cells. In the Spo11 pulling down ChIP-seq experiment, sequence reads from Spo11 bound or MNase-resistant oligos account for only 19.13% and 21.96% at the DSB regions or 7.1% and 6.7% of total sequence reads at the control regions of the diploid and tetraploid yeast, respectively. In the MNase-seq experiments, these figures are 3.9% and 3.4% at the DSB regions or 5.0% and 5.1% at the control regions (supplementary table 7, Supplementary Material online). Additionally, a markedly high level of positive correlation in ChIP-seq or MNase-seq data was observed between the diploid and tetraploid yeasts. These suggest that the RPKM normalization would be recognized appropriate for comparing the sequence data between diploid and tetraploid yeasts in the present study

## Discussion

This study reports the first comprehensive survey of the ploidy-driven change in MRF in *S. cerevisiae*. It demonstrates that a significant increase in MRF occurred after genome duplication in all examined regions covering both recombination hot and cold spots on all 16 yeast chromosomes. Given that formation of COs is a prerequisite for recombination, we assayed profile of COs on the all 16 chromosomes of the diploid and tetraploid yeast genomes using genome sequencing and found that rate of COs is, on single chromosome basis, highly significantly higher in the autotetraploid genome than in the corresponding diploid genome. It should be acknowledged that crossing over and gene conversion are associated in meiosis. Gene conversion and crossing over may be regulated independently, and crossing over may involve interference but gene conversion may not (Engebrecht et al. 1990). The present tetrad sequence data should allow to work out the rate of gene conversion in addition to the rate of COs. However, tetraploid tetrads are diploids and how to estimate gene conversion from the tetraploid tetrad sequence data remains an open question for further exploitation.

To explain the ploidy-driven increase in MRF, we compared level of RI between diploid and tetraploid genomes and observed that RI is a clearly weakened in the genomic regions investigated. Additionally, a Spo11 pulled down DNA sequencing experiment indicates a significantly higher frequency of single-chromosome-based DSB in the tetraploid genome than in the corresponding diploid counterpart at the 443 commonly shared and previously identified sites in meiosis of both the diploid and tetraploid yeasts. The observation from the ChIP-seq data is further verified independently by the Southern blotting assay and return-to-growth experiment. DNA sequence data collected at MNase-resistant mononucleosomes shows that the tetraploid chromosomes are comparatively less wrapped by nucleosomes than the diploid chromosomes, having provided the structural basis for the difference in DSB frequency between the diploid and tetraploid genomes. Although there has yet been mechanistic explanation for association of genome duplication with nucleosome occupancy to our best knowledge, it has been well established that genome duplication is in parallel with the dynamic alteration in genome structure and functionality such as genome-wide gene expression and phenotypic change (Song et al. 1995; Comai 2005). On the other hand, nucleosome occupancy is partially encoded by intrinsic antinucleosomal DNA sequences as well as by binding sites for *trans*-acting factors that can evict nucleosomes in yeast (Jiang and Pugh 2009). Moreover, binding site gain or loss events at nucleosome-depleted regions in yeast genome may cause more expression differences than those in nucleosome-occupied regions (Swamy et al. 2011). Thus, the present study could stimulate further study to elucidate the molecular mechanism underpinning genome duplication-associated change in nucleosome occupancy.

Although it has been theoretically predicted that meiotic recombination would be more frequent in tetraploids than in diploids (Sved 1964), there are only a few experimental studies of limited scale by far performed to test this theoretical prediction (Oram 1959; Welch 1962). More recently, Pecinka et al. (2011) compared frequency of meiotic recombination of two fluorescent makers between diploid and artificially synthesized tetraploid in *Arabidopsis*, and concluded a higher recombination frequency at the marked chromosomal interval in tetraploid than in diploid. In fact, appropriate statistical methods for linkage analysis under a tetrasomic inheritance model are essential for statistically appropriate evaluation of genetic recombination frequency (Wang and Luo 2012). One of other important distinctions of the present study from its rivals in the literature is the development of statistically appropriate methods for modeling and analyzing the experimental data under a tetrasomic inheritance basis. This not only enables the data-specific analysis in the present study but also provides useful analytical tools for the tetrasomic linkage and genetic analyses with other tetraploid species.

Meiotic recombination is the major mechanism for genetic variation blocked within individual genomes to be released for natural and artificial selection, and thus is recognized as one of major driving factors for the evolution and/or speciation (Otto and Barton 1997) as well as for breaking limits of artificial selection (Hill and Robertson 2007). On the other hand, polyploidy has played an important role in the evolution of eukaryotes, particularly flowering plants, with 30–80% of angiosperms being currently polyploid, and the rest existing as paleopolyploids, having evolved from and/or reverted to a diploid state over evolutionary time (Otto and Whitton 2000). It has been well established that a genome in polyploidy has three distinct advantages. Firstly, a high level of heterozygosity maintained enables polyploids to be more vigorous than their diploid progenitors. Secondly, a larger number of segregating alleles shield the polyploid genome from deleterious mutation. Thirdly, if not finally, most polyploids may reproduce asexually and thus can be propagated much efficiently into large populations (Comai 2005). In addition to these, the present study contributes a significant feature to polyploidization through genome duplication, that is, the significantly increased MRF. Thus, the present study, on one hand, fills a gap between plolyploidization and evolution of species via the significant effect of genome duplication on the genome’s recombination frequency, and on the other, opens an opportunity for artificial manipulation of meiotic recombination through polyploidization breeding, as has been seeking for by plant scientists and breeders for many years (Henderson 2012).

## Materials and Methods

All strains used in this study were isogenic to s288c and SK1. Autotetraploids were constructed through fusion between the mating-type switched a/a and α/α diploids, which were induced and screened from the normal a/α diploids transformed with pTetra plasmid, as described in our previous study (Fang et al. 2018). Fluorescent and antibiotic markers were generated by using of polymerase chain reaction, transformed into strains through the PEG-LiAC method, and inserted into predesigned loci of chromosomes. Unless specified, both wild-type and genetically modified strains were grown in the standard rich medium YPD (1% yeast extract, 2% polypeptone, 2% glucose, plus 2% agar if necessary). Meiosis was induced in the SPM medium (1% potassium acetate, plus 2% agar if necessary). Mating-type switch was stimulated in YPGal (1% yeast extract, 2% polypeptone, 2% galactose, 2% ranffinose). Test strains were synchronized at the same meiosis stage in the YPG plates (3% glycerol, 2% polypeptone, 1% yeast extract, 2% agar) and SPS medium (1% potassium acetate, 1% w/v polypeptone, 0.5% yeast extract, 0.17% yeast nitrogen base with ammonium sulfate and without amino acids, 0.5% ammonium sulfate, 0.05 M potassium biphtalate, 2 drops per liter antifoam, pH to 5.5 with 10 N KOH). The ChIP-seq and MNase-seq assays were implemented according to the classic protocol, but with minor modification (Prieler et al. 2005; Kaplan et al. 2009). Detailed description of all experimental materials and methods used can be found in Supplementary Material online.

### Statistical Method 1: Statistical Modeling and Analysis of the Fluorescent Maker Data

To model and analyze the fluorescent marker data collected from the diploid and autotetraploid segregating populations, we proposed here the probabilistic models for modeling the marker data and statistical methods for analyzing the data sets. The model and statistical methods have properly accounted for the key features of disomic and tetrasomic inheritance of gene segregation and recombination and solved properly the challenges in the statistical analysis. Our formulation here focuses on the autotetraploids. We considered a general tetraploid genotype at two loci, A_1_A_2_A_3_A_4_/B_1_B_2_B_3_B_4_, and assumed that the alleles at the two loci are linked with a recombination frequency *r*, and the coefficient of double reduction at locus A is *a*. We have worked out the probability distribution of 136 possible diploid gamete genotypes generated by the autotetraploid individual genotype in term of *r* and *a* (table 1 in Luo et al. 2004). In the present context, we worked out the probability distribution of ten possible diploid gamete genotypes generated by the parental genotype, GBBB/RCCC by setting A_1_ = G, B_1_ = R, A_2_ = A_3_ = A_4_ = B, and B_2_ = B_3_ = B_4_ = C and summing up the corresponding genotype frequencies for the same gamete genotype. Table 1 lists the probability distribution for the ten gamete genotypes $g_{i}(\alpha ,r)$ (*i *=* *1, 2, … , 10), which describes the probability distribution of the genotypes in the yeast tetrad spore population created in the study. However, genotypes of the diploid spores cannot be directly observable but the spores can be grouped according to four possible fluorescent phenotype classes (yellow, green, red, and black). Probabilities of the phenotype groups are given as

(1) $$\begin{array}{c} f_{y}(\alpha ,r)=g_{1}(\alpha ,r)+g_{2}(\alpha ,r)+g_{3}(\alpha ,r)+g_{4}(\alpha ,r)+g_{5}(\alpha ,r)\\ =\frac{1}{12}\left[2(3-3r+r^{2})-\alpha (3-6r+5r^{2})\right] \end{array}$$

(2) $$f_{g}(\alpha ,r)=g_{6}(\alpha ,r)+g_{7}(\alpha ,r)=\frac{r}{12}\left[6-2r-\alpha (6-5r)\right].$$

(3) $$f_{r}(\alpha ,r)=g_{8}(\alpha ,r)+g_{9}(\alpha ,r)=\frac{1}{36}(2+\alpha )r(6-r).$$

(4) $$f_{b}(\alpha ,r)=g_{10}(\alpha ,r)=\frac{1}{36}(2+\alpha ){\left(3-r\right)}^{2}.$$

Let $n_{i}$ denote by the number of diploid spores with the *i*th phenotype (*i *=* *1, 2, 3, 4 corresponding to *y*, *g, r*, *b*, respectively) and $n=n_{1}+n_{2}+n_{3}+n_{4}$. The log-likelihood of the model parameters, *α* and *r*, given the observed *n_i_*’s, is given by

(5) $$L(a,r|\;n_{i})=\sum_{i=1}^{4}n_{i}\;\text{log}[f_{i}(a,r)].$$

Because *a* indicates the coefficient of double reduction at the locus nearer to the centromere, information about segregation of alleles at the locus is sufficient to estimate the parameter (Alani et al. 1990). To work out the double reduction parameter, we set *r *=* *0 in the likelihood function (5), solved the equation for *α*, and obtained the maximum likelihood estimate (MLE)

$(n_{1}+n_{2})\partial \{\;\text{log}[f_{1}(\alpha ,0)+f_{2}(\alpha ,0)]\}/\partial \alpha +(n_{3}+n_{4})\partial \{\;\text{log}[f_{3}(\alpha ,0)+f_{4}(\alpha ,0)]\}/\partial \alpha =0$     (6)

$\hat{\alpha }=2(n_{3}+n_{4}-n_{1}-n_{2})/n$.   (7)

The asymptotic sampling variance of the MLE can be calculated according to the Fisher’s information metric from

(8) $$\begin{array}{c} -{\left[(n_{1}+n_{2})\partial^{2}\{\;\text{log}[f_{1}(\alpha ,0)+f_{2}(\alpha ,0)]\}/\partial \alpha^{2}+(n_{3}+n_{2})\partial^{2}\{\;\text{log}[f_{3}(\alpha ,0)+f_{4}(\alpha ,0)]\}/\partial \alpha^{2}\right]}_{\alpha =\hat{\alpha }}^{-1}\\ =16\times (n_{1}+n_{2})(n_{3}+n/n. \end{array}$$

The formulation can be modified by exchanging between $f_{1}(\alpha ,0)$ and $f_{2}(\alpha ,0)$ and also exchanging between $n_{2}$ and $n_{3}$ to calculate $\hat{\alpha }$, the coefficient of double reduction at the red fluorescent locus, which is distal to the centromere in the model.

We calculated the MLE of recombination frequency *r* directly from solving $\partial L(\hat{a},r|\;n_{i})/\partial r=0$, which is equivalent to a polynomial equation of grade 5 and has no a simple and close form for the solution. The equation can be numerically solved and the root within in the range 0.0 and 0.75 was taken as the MLE $\hat{r}$. The asymptotic sampling variance for $\hat{r}$ can be calculated from ${-1/\left[\partial^{2}L(\hat{a},r|\;n_{i})/\partial r^{2}\right]}_{r=\hat{r}}$.

istribution of phenotype at the single marker locus is given by $f_{R|G}(\alpha )=(4-\alpha )/12$ and $f_{B}(\alpha )=(8+\alpha )/12$ for the individuals with and without carrying the fluorescent marker, respectively. If the number of the two groups of individuals is denoted by *n*_1_ and *n*_2_, respectively,

### Statistical Method 2: Predicting the Average Number of COs from Tetraploid Gamete Data

Consider a marker interval and let *α* be the coefficient of double reduction at the flanking marker locus, which is nearer to the centromere, and *p* represents the probability of 1 CO in the marker interval. We focused here gametogenesis of an autotetraploid individual with the genotype, *AB/ab/ab/ab*, with A and B corresponding to s288c (SK1) alleles, and *a* and *b* to SK1 (or s288c) alleles in the autotetraploid strain s288c/SK1/SK1/SK1 (or SK1/s288c/s288c/s288c). We considered the CO occurring between all possible nonsister chromatids and all possible configurations of diploid gamete generation under a tetrasomic model, and worked out distribution of phenotype of five possible tetrads at the two marker loci in term of *α* and *p*, which was listed as supplementary table 4, Supplementary Material online. In the distribution, a tetrad phenotype was presented as two sequential integers representing two chromosomes. A nonzero integer in the sequence represented the number of A or B alleles and the four integers referred to the four spores.

For a sample of *n* tetrads, let *n_i_* (*i *=* *1, 2, … , 5) be the number of tetrads with the *i*th marker phenotype. The log-likelihood of the model parameters, *α* and *p*, given the observed *n_i_* is given by

(9) $$\begin{array}{c} L(\alpha ,p|n_{i})\propto \sum_{i=1}^{5}n_{i}\;\text{log}(f_{i})=n_{1}\;\text{log}\;\{\alpha (1-p/2)\}+n_{2}\;\text{log}\;\{(1-\alpha )(1-5p/12)\}+n_{3}\;\text{log}\;\{\alpha p/2\}\\ +n_{4}\;\text{log}\;\{(1-\alpha )p/12\}+n_{5}\;\text{log}\;\{(1-\alpha )p/3\} \end{array}$$

Differentiating the above and solving the normal equation led to the maximum likelihood estimate of *p* as

(10) $$\hat{p}=\left(11n-5n_{1}-6n_{2}\pm \sqrt{\Delta }\right)/5n$$

with $\Delta =n^{2}+2n(5n_{1}-6n_{2})+{\left(5n_{1}+6n_{2}\right)}^{2}$. Choice of the alternatives is feasible because a meaningful estimate of *p* must fall in [0, 1]. Sum of estimates of *p* over all marker intervals on a chromosome gives an estimate of expected number of COs of that chromosome.

## Supplementary Material

Supplementary data are available at *Molecular Biology and Evolution* online.

## Supplementary Material

msaa219_Supplementary_Data
