# Supplementary material for: Genome Duplication Increases Meiotic Recombination Frequency: A Saccharomyces cerevisiae Model
Source: Mol Biol Evol. 2020 Sep 8;38(3):777–87. doi: 10.1093/molbev/msaa219 (PMC7947769; doi:10.1093/molbev/msaa219)
Supplement: msaa219_Supplementary_Data [file msaa219_supplementary_data.docx]

**Supporting Information**

**
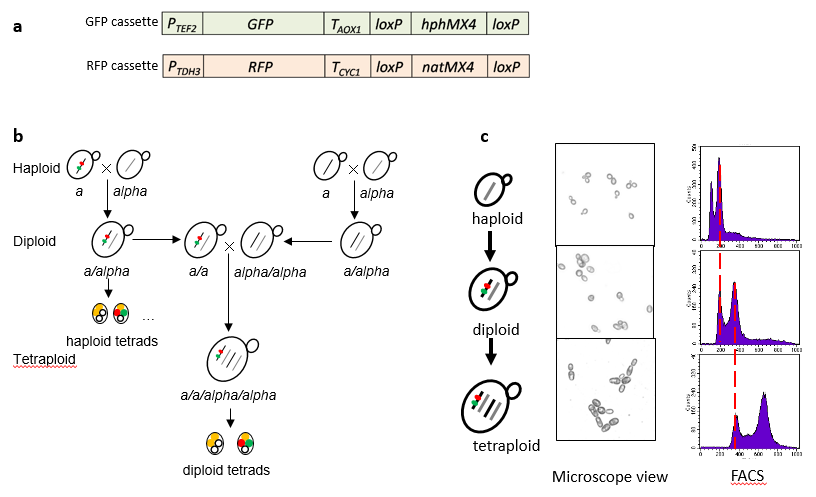
**

**Figure S1**. (**a**) construct of fluorescent cassettes carrying *GFP* and *RFP*. (**b**) Diagrammatic illustration of breeding program for creating diploid and autotetraploid yeast cells from a laboratory haploid strain. (**c**) FACS (fluorescence-activated cell sorting) profiles of genomic DNA content of yeast cells at haploid, diploid and tetraploid levels.

| **Supplementary Table S1**. Physical location of the GFP and RFP cassettes and the centromere in each of the sixteen yeast chromosomes as well as physical distance between the fluorescent genes, which may locate within previously identified recombination hot (red) or cold (blue) spots. | | | | | | | | | | | |
| --- | --- | --- | --- | --- | --- | --- | --- | --- | --- | --- | --- |
| Chr. | Length(kb) | CEN | |  | RFP | |  | GFP | | Distance | Hot/cold spot |
|  |  | start | end |  | Between ORF | Location |  | Between ORF | Location |  |  |
| I | 230218 | 151465 | 151582 |  | YAL027W/YAL028W | 94527 |  | YAL040C/YAL041W | 65575 | 28953 | YAL038W |
| II | 813184 | 238207 | 238323 |  | YBR149W/YBR150C | 541090 |  | YBR181C/YBR182C | 593277 | 52188 |  |
| III | 316620 | 114385 | 114501 |  | YCL018W | 92419 |  | YCL030C | 65934 | 26486 | YCL030C |
| IV | 1531933 | 449711 | 449821 |  | YDR216W/YDR217C | 899252 |  | YDR233C/YDR234W | 930819 | 31568 |  |
| V | 576874 | 151987 | 152104 |  | YER090W/YER091C | 339666 |  | YER109C/YER110C | 378289 | 38624 |  |
| VI | 270161 | 148510 | 148627 |  | YFR020W/YFR021W | 193691 |  | YFR039C/YFR040W | 233793 | 40103 | YFR025C |
| VII | 1090940 | 497038 | 496920 |  | YGL173C/YGL174W | 175425 |  | YGL147C/YGL148W | 227613 | 52189 |  |
| VIII | 562643 | 105703 | 105586 |  | YHR002W/YHR003C | 108308 |  | YHR025W/YHR026W | 160583 | 52276 | YHR018C |
| IX | 439888 | 355629 | 355745 |  | YIL096C/YIL097W | 182050 |  | YIL116W/YIL117C | 142447 | 39604 |  |
| X | 745751 | 436425 | 436307 |  | YJR061W/YJR062C | 553386 |  | YJR084W/YJR085C | 585080 | 31695 |  |
| XI | 666816 | 440246 | 440129 |  | YKL198C/YKL201C | 67988 |  | YKL215C/YKL216W | 26546 | 41443 |  |
| XII | 1078177 | 150947 | 150828 |  | YLR148W/YLR149C | 437619 |  | YLR162W-A/YLR163C | 490808 | 53190 | rDNA |
| XIII | 924431 | 268031 | 268149 |  | YMR279C/YMR280C | 826778 |  | YMR299C/YMR300W | 865463 | 38686 |  |
| XIV | 784333 | 628758 | 628875 |  | YNL322C/YNL323W | 33284 |  | YNL185C/YNL186W | 292057 | 258774 |  |
| XV | 1091291 | 326702 | 326584 |  | YOR010C/YOR011W | 347936 |  | YOR027W/YOR028C | 383077 | 35142 |  |
| XVI | 948066 | 555957 | 556073 |  | YPL014W/YPL015C | 527317 |  | YPL037C/YPL038W | 481227 | 46091 |  |

**Supplementary Table 2** The number of tetrads scored for phenotype of the two florescent makers locating on each of sixteen yeast chromosomes and segregating in diploid or autotetraploid populations.

| Chromosome | Ploidy | Phenotype of tetrads | | | | | | | | |  |
| --- | --- | --- | --- | --- | --- | --- | --- | --- | --- | --- | --- |
|  |  | YYBB | YGRB | GGRR | YBBB | YGBB | YRBB | YYGB | YYRB | Total | |
| I | Diploids | 359 | 249 | 6 | 2 | 4 | 2 | 0 | 6 | 628 | |
|  | Tetraploids | 549 | 272 | 8 | 18 | 14 | 16 | 2 | 5 | 884 | |
| II | Diploids | 465 | 138 | 1 | 0 | 2 | 0 | 0 | 1 | 607 | |
|  | Tetraploids | 451 | 147 | 3 | 17 | 14 | 11 | 0 | 1 | 644 | |
| III | Diploids | 600 | 151 | 2 | 0 | 0 | 2 | 0 | 1 | 756 | |
|  | Tetraploids | 601 | 194 | 2 | 3 | 4 | 19 | 3 | 3 | 829 | |
| IV | Diploids | 517 | 94 | 0 | 0 | 1 | 0 | 9 | 0 | 621 | |
|  | Tetraploids | 518 | 97 | 1 | 34 | 11 | 16 | 7 | 0 | 684 | |
| V | Diploids | 482 | 117 | 1 | 0 | 1 | 0 | 3 | 1 | 605 | |
|  | Tetraploids | 473 | 133 | 0 | 21 | 7 | 12 | 4 | 0 | 650 | |
| VI | Diploids | 296 | 327 | 11 | 0 | 2 | 7 | 3 | 2 | 648 | |
|  | Tetraploids | 241 | 377 | 21 | 4 | 6 | 24 | 4 | 1 | 678 | |
| VII 25℃ | Diploids | 600 | 272 | 4 | 1 | 1 | 0 | 2 | 0 | 880 | |
|  | Tetraploids | 1068 | 566 | 15 | 66 | 60 | 49 | 2 | 2 | 1828 | |
| VII 30℃ | Diploids | 600 | 313 | 4 | 0 | 1 | 2 | 2 | 1 | 923 | |
|  | Tetraploids | 1203 | 706 | 16 | 85 | 62 | 51 | 2 | 4 | 2129 | |
| VIII | Diploids | 310 | 308 | 7 | 1 | 1 | 0 | 4 | 0 | 631 | |
|  | Tetraploids | 297 | 311 | 6 | 0 | 0 | 11 | 7 | 0 | 632 | |
| IX | Diploids | 373 | 227 | 2 | 0 | 0 | 0 | 0 | 0 | 602 | |
|  | Tetraploids | 406 | 232 | 2 | 9 | 7 | 15 | 1 | 0 | 672 | |
| X | Diploids | 410 | 208 | 6 | 0 | 1 | 0 | 0 | 1 | 626 | |
|  | Tetraploids | 395 | 212 | 3 | 15 | 7 | 17 | 0 | 0 | 649 | |
| XI | Diploids | 419 | 193 | 3 | 0 | 2 | 6 | 0 | 5 | 628 | |
|  | Tetraploids | 454 | 153 | 1 | 18 | 15 | 17 | 1 | 7 | 666 | |
| XII | Diploids | 600 | 88 | 0 | 0 | 0 | 1 | 0 | 0 | 689 | |
|  | Tetraploids | 600 | 100 | 5 | 28 | 8 | 12 | 6 | 0 | 759 | |
| XIII | Diploids | 390 | 225 | 3 | 0 | 4 | 2 | 0 | 0 | 624 | |
|  | Tetraploids | 420 | 192 | 4 | 30 | 26 | 27 | 0 | 1 | 700 | |
| XIV | Diploids | 164 | 603 | 115 | 0 | 0 | 11 | 0 | 2 | 895 | |
|  | Tetraploids | 208 | 600 | 108 | 20 | 49 | 39 | 0 | 2 | 1026 | |
| XV | Diploids | 563 | 40 | 0 | 0 | 0 | 0 | 0 | 0 | 603 | |
|  | Tetraploids | 544 | 62 | 0 | 2 | 0 | 4 | 0 | 3 | 615 | |
| XVI | Diploids | 376 | 245 | 2 | 1 | 1 | 1 | 0 | 3 | 629 | |
|  | Tetraploids | 364 | 249 | 0 | 6 | 1 | 11 | 4 | 6 | 641 | |
| Total | Diploids | 7524 | 3798 | 167 | 5 | 21 | 34 | 23 | 23 | 11,595 | |
|  | Tetraploids | 8792 | 4603 | 195 | 376 | 291 | 351 | 43 | 35 | 14,686 | |

| **Supplementary Table 3** Physical locations of the inserted anti-biotic markers *NatMX4*, *hphMX4* and *KanMX4* on the yeast chromosomes III, VI and VIII. | | | | |
| --- | --- | --- | --- | --- |
| Chromosomes | | Chr. III | Chr. VI | Chr. VIII |
| Chr. Length (bps) | | 316620 | 270161 | 562643 |
| Chr. Location | | 114385 | 148627 | 105586 |
| *NatMX4* | ORF | YCL018W | YFR020W/YFR021W | YHR002W/YHR003C |
|  | location | 92419 | 193691 | 108308 |
| *hphMX4* | ORF | YCL030C | YFR039C-040W | YHR025W/026W |
|  | location | 65934 | 233793 | 160583 |
| *KanMX4* | ORF | YCL073C/074W | YFR056C/YFR057W | YHR047C/YHR048W |
|  | location | 6986 | 270856 | 197925 |

**Supplementary Table 4** Distribution of two loci phenotype data of tetrads generated from a parent strain *AB/ab/ab/ab.*

| 0 crossover | | | | | | | |
| --- | --- | --- | --- | --- | --- | --- | --- |
| Double Reduction | Tetrad | | | | Scoring on tetrad | Category | probability |
|  | gamete 1 | gamete 2 | gamete 3 | gamete 4 |  |  |  |
| 1 | $AA/BB$ | $aa/bb$ | $aa/bb$ | $aa/bb$ | $2000/2000$ | 1 | $\left(1-p\right)\alpha$ |
| 0 | $Aa/Bb$ | $Aa/Bb$ | $aa/bb$ | $aa/bb$ | $1100/1100$ | 2 | $\left(1-\alpha\right)\left(1-p\right)$ |
| 1 crossover | | | | | | | |
| Double Reduction | Tetrad | | | | Scoring on tetrad | Category | probability |
|  | gamete 1 | gamete 2 | gamete 3 | gamete 4 |  |  |  |
| 1 | $AA/Bb$ | $aa/Bb$ | $aa/bb$ | $aa/bb$ | $2000/1100$ | 3 | $\alpha p/2$ |
|  | $AA/BB$ | $aa/bb$ | $aa/bb$ | $aa/bb$ | $2000/2000$ | 1 | $\alpha p/2$ |
| 0 | $Aa/bB$ | $Aa/Bb$ | $aa/bb$ | $aa/bb$ | $1100/1100$ | 2 | $p'\left(1-\alpha\right)/12$ |
|  | $Aa/bb$ | $Aa/BB$ | $aa/bb$ | $aa/bb$ | $1100/0200$ | 4 | $p'\left(1-\alpha\right)/12$ |
|  | $Aa/bb$ | $Aa/Bb$ | $aa/Bb$ | $aa/bb$ | $1100/0110$ | 5 | $p'\left(1-\alpha\right)/3$ |
|  | $Aa/Bb$ | $Aa/Bb$ | $aa/bb$ | $aa/bb$ | $1100/1100$ | 2 | $p'\left(1-\alpha\right)/2$ |

In the column of double reduction, 1(0) indicates the presence (absence) of double reduction on the first marker locus of the two loci interval. Scoring on the four spores of the two loci tetrad follows the rules: 2 represents *AA* (*BB*), 1 represents *Aa*(*Bb*) and 0 represent *aa*(*bb*). Here $\alpha$ is the coefficient of double reduction on locus A and $p'$ is the probability of a crossover event within the marker interval.

**Supplementary Table 5** Primer sequences used for PCR to construct the fluorescent DNA fragments used in yeast transformations

| **Primers for RFP cassette insertion** | |
| --- | --- |
| ChrI_RFP_F | AAAATAGTATTTGTATATCAAAAAATGATCCTGTGATTTTTAGCTGAAGCTTCGTACGC |
| ChrII_RFP_F | GTTCGCTTCTTAATCTTGTCAAAAACAAGATATTGTGTAATAGCTGAAGCTTCGTACGC |
| ChrIII_RFP_F | CAGCAATATATATATATATATTTCAAGGATATACCATTCTAAGCTGAAGCTTCGTACGC |
| ChrIV_RFP_F | TCTCGTTATTGCTCCTTATTTTGGTCCATTTTATATTAAATAGCTGAAGCTTCGTACGC |
| ChrV_RFP_F | TATAGGAAATTTTAGAAAACGAATGTTGAATGCGTTATAAAAGCTGAAGCTTCGTACGC |
| ChrVI_RFP_F | TAGGAGTGCATATAAATGGACACTTGGATTCTTCATTAGATAGCTGAAGCTTCGTACGC |
| ChrVII_RFP_F | CTGAGACCTATATAAAGAGGGGTATTTGTATTTAATCGTTGAGCTGAAGCTTCGTACGC |
| ChrVIII_RFP_F | GGATCATAAAAGGTATAAAAAGTAATTGATCCATACAACATAGCTGAAGCTTCGTACGC |
| ChrIX_RFP_F | TGATGAAAGAACTTTCATTTTCCCTAATTCTTATATAGCGTAGCTGAAGCTTCGTACGC |
| ChrX_RFP_F | GATAATAACAAAAACCATTATTTATTCATTCTTGCCTCACTAGCTGAAGCTTCGTACGC |
| ChrXI_RFP_F | TGCCAAGAAACTCCGCTAAACTAACATCGCGATGCAATGGGAGCTGAAGCTTCGTACGC |
| ChrXII_RFP_F | ATAAAACGTAAAATTATCGGGGAAAAACCTTGTATTTTCAAAGCTGAAGCTTCGTACGC |
| ChrXIII_RFP_F | ATTAATGGAACTACATTTCAGATTAGGAAATTTTTTTGCTAAGCTGAAGCTTCGTACGC |
| ChrXIV_RFP_F | AAAAGGAGTGATGGTTTTCTTTATTTTTAACCCTGTAAAATAGCTGAAGCTTCGTACGC |
| ChrXV_RFP_F | AAAAAGATCCTATGTGAGTTAACAAGAAACTTAAAACAATCAGCTGAAGCTTCGTACGC |
| ChrXVI_RFP_F | GGATAGGCACGTCAAACTCATTAGGTCTCATCAAGAACTAGAGCTGAAGCTTCGTACGC |
| ChrI_RFP_R | AGAAGAAGCACATTTTTACATTTATACGTTACATATGAGCATAGGCCACTAGTGGATC |
| ChrII_RFP_R | TCAAATCACGTATGAAAAACATATTGTTTACTTGAGGCGGCATAGGCCACTAGTGGATC |
| ChrIII_RFP_R | AAAGTTTATGTACAAATATCATAAAAAAAGAGAATCTTTGCATAGGCCACTAGTGGATC |
| ChrIV_RFP_R | TATGAGGTATAAGATGAAAAATGTTAAACATGATACAGGGCATAGGCCACTAGTGGATC |
| ChrV_RFP_R | TTTTTATTATATTTTTATATACACGTACATTCTGTATTAGCATAGGCCACTAGTGGATC |
| ChrVI_RFP_R | ATCCTACAAAAGAAATTGCTAAGATCTGATACAGTGACCGCATAGGCCACTAGTGGATC |
| ChrVII_RFP_R | AAATACCCTTTGACAATCCCCATTTGTTATAAGCTTTTTGCATAGGCCACTAGTGGATC |
| ChrVIII_RFP_R | TTTTAGTAAATATTCTGAAAATAGAGGGGTAAATATTTAGCATAGGCCACTAGTGGATC |
| ChrIX_RFP_R | AAGATTTGTCCAACTTTCACAACTATTTTACAGATACTTGCATAGGCCACTAGTGGATC |
| ChrX_RFP_R | ATTTATGTTATTGAAATGTCGCCGTAAGGGCCGAATTTAGCATAGGCCACTAGTGGATC |
| ChrXI_RFP_R | CTTTGCGCCGGTTTTCATTTTCTTCCACGGAATACCAAGGCATAGGCCACTAGTGGATC |
| ChrXII_RFP_R | ACATATATTGTGATTTTAATTCACCCGTTTTATTACTCTGCATAGGCCACTAGTGGATC |
| ChrXIII_RFP_R | CGCTGTCAGATGGTCCTCCGGTAACGTAACCATGGGAACGCATAGGCCACTAGTGGATC |
| ChrXIV_RFP_R | TATTAACGGTTTTAATTTGGTTCTTATAAATTTTTATTCGCATAGGCCACTAGTGGATC |
| ChrXV_RFP_R | CATATTCTGGTCTAAAAAAATTATACTAGGCAGGTAATGGCATAGGCCACTAGTGGATC |
| ChrXVI_RFP_R | ACTTTGTTTAAAGTTCGGAGTTCAAGCGTCATTTCTACAGCATAGGCCACTAGTGGATC |
| **Primers for GFP cassette insertion** | |
| ChrI_GFP_F | ATTATAAAAACGCACAACCTAAAATACGATTACTATCGTTTAGCTGAAGCTTCGTACGC |
| ChrII_GFP_F | TTTTTGTGCACTCAATCGATCAAAACAATAGAGACTGCCCTAGCTGAAGCTTCGTACGC |
| ChrIII_GFP_F | TAATAGTAATACAATAGTTTACAAAATTTTTTTTCTGAATAAGCTGAAGCTTCGTACGC |
| ChrIV_GFP_F | ATTTCGTGTCAAGTGAGCAGAATGGAGCGATTTGGAAAAAAAGCTGAAGCTTCGTACGC |
| ChrV_GFP_F | TCAGGTCATCTTGTACTTGTTCTAATGAAAGAATCACGGCAAGCTGAAGCTTCGTACGC |
| ChrVI_GFP_F | ACAAAATGAGTAGGGTTAATTTAATTTTTAAAGAGGGCATTAGCTGAAGCTTCGTACGC |
| ChrVII_GFP_F | GCATGTATATGTATTAGTGCACGTAATAAAACGGCAACAAAAGCTGAAGCTTCGTACGC |
| ChrVIII_GFP_F | ATACTATTGTAGACCATATACATACTGTAATTGACCGTTAAAGCTGAAGCTTCGTACGC |
| ChrIX_GFP_F | AACTTTGCAAAAATAAAGGGTAAATGGTTAAAAATTGTTATAGCTGAAGCTTCGTACGC |
| ChrX_GFP_F | AAAGAAAGTTTGTTCATAAAAGAATATCATATTATATATATAGCTGAAGCTTCGTACGC |
| ChrXI_GFP_F | TATTCGTTAATTTCTTCACTTTTTTCCCCAAATATTAAACAAGCTGAAGCTTCGTACGC |
| ChrXII_GFP_F | CCTGATGAGTTCTGCATCTGTCCTATCTACCTATTTTACCGAGCTGAAGCTTCGTACGC |
| ChrXIII_GFP_F | TTATACATAATTATCTTTGTATTCTGACCTAAACTAAATATAGCTGAAGCTTCGTACGC |
| ChrXIV_GFP_F | AGAAGCGTTTACTCAGAATAGCTTCAGTACTACCGAGCTTTAGCTGAAGCTTCGTACGC |
| ChrXV_GFP_F | AAATTTCTGGCCGGAACTCTCCTTAGAAACAAAAAAAATTAAGCTGAAGCTTCGTACGC |
| ChrXVI_GFP_F | TGCTGCACACGTGAAATGATGAACTTGTACGTCACCACTTTAGCTGAAGCTTCGTACGC |
| ChrI_GFP_R | AAAAAAATGTATAATGTGACTAGAGGAAGTAAGGAGAAAGCATAGGCCACTAGTGGATC |
| ChrII_GFP_R | ATGAGATAATTTTTTACATAATTATGTTGAGAAGTTAACGCATAGGCCACTAGTGGATC |
| ChrIII_GFP_R | TTATTTTCGTTAGTGTTCGGTTTCCAAGTTAGAAATAATGCATAGGCCACTAGTGGATC |
| ChrIV_GFP_R | CTCGAGCAAAATCCCGGGGGAAAAAACAATAAAAATTTTGCATAGGCCACTAGTGGATC |
| ChrV_GFP_R | TGTACAAGACCAATATCCGGGGCCCGCTAATTAGTAACGGCATAGGCCACTAGTGGATC |
| ChrVI_GFP_R | TAATTACCAGCCCTATAGTTGTCTTTCATTTTTTAGATAGCATAGGCCACTAGTGGATC |
| ChrVII_GFP_R | TACGAAAATAAAAGTACCGATAATATGTCAACTTTTTGTGCATAGGCCACTAGTGGATC |
| ChrVIII_GFP_R | TAGAAAAGACGGGTAACGGAAGAAGACTCAGCATAATAAGCATAGGCCACTAGTGGATC |
| ChrIX_GFP_R | CCAGGAAAGGTCTCAATATAACCGGTCACCTTATTTATGGCATAGGCCACTAGTGGATC |
| ChrX_GFP_R | AGAAGGAAGAAGACGCACGATATATATACGACCCTATATGCATAGGCCACTAGTGGATC |
| ChrXI_GFP_R | ATAGGAACCAGAAAATAATGTTGAAGAAATGAAGCCATCGCATAGGCCACTAGTGGATC |
| ChrXII_GFP_R | TCCTAAAAAGATTGATTACGCAGACAGTATTTGTGTTACGCATAGGCCACTAGTGGATC |
| ChrXIII_GFP_R | GCTTTTATTTTTTTTTTTAATACGTGCATATATATATATGCATAGGCCACTAGTGGATC |
| ChrXIV_GFP_R | TATATAAGTTTTAAATTCTCTTTCTCCCGTTATGTTAGCGCATAGGCCACTAGTGGATC |
| ChrXV_GFP_R | TGTAATGAATATTGTTGACGTAAAGTTGTGCCAATTACGGCATAGGCCACTAGTGGATC |
| ChrXVI_GFP_R | TAGTGACATGTTCTCTACTTTTGCCAATTTTTTGCGCACGCATAGGCCACTAGTGGATC |

**Supplementary Table 6** Crossover counts per meiosis

| Chr | Arm | Arm Length(kb) | Diploids | | | | | Autotetraploids | | | | | |
| --- | --- | --- | --- | --- | --- | --- | --- | --- | --- | --- | --- | --- | --- |
|  |  |  | hs1 | hs2 | hs3 | hs4 | hs5 | hhhs1 | hhhs2 | hhhs3 | hsss1 | hsss2 | hsss3 |
| I | L | 151 | 1 | 2 | 2 | 3 | 2 | 1 | 2 | 2 | 1 | 0 | 1 |
|  | R | 79 | 0 | 0 | 0 | 0 | 1 | 1 | 1 | 0 | 1 | 0 | 1 |
| II | L | 238 | 1 | 1 | 1 | 0 | 2 | 0 | 2 | 1 | 0 | 1 | 2 |
|  | R | 575 | 3 | 3 | 3 | 4 | 2 | 6 | 4 | 6 | 3 | 3 | 1 |
| III | L | 114 | 2 | 0 | 0 | 1 | 1 | 1 | 1 | 1 | 0 | 1 | 1 |
|  | R | 202 | 2 | 0 | 2 | 1 | 2 | 2 | 2 | 1 | 1 | 3 | 2 |
| IV | L | 450 | 2 | 4 | 2 | 4 | 3 | 3 | 4 | 6 | 4 | 3 | 4 |
|  | R | 1082 | 4 | 5 | 6 | 6 | 7 | 4 | 5 | 5 | 10 | 8 | 8 |
| V | L | 152 | 1 | 1 | 1 | 1 | 1 | 1 | 0 | 0 | 1 | 0 | 2 |
|  | R | 425 | 3 | 4 | 2 | 1 | 2 | 4 | 6 | 2 | 1 | 5 | 5 |
| VI | L | 149 | 0 | 2 | 1 | 1 | 1 | 2 | 2 | 0 | 0 | 3 | 0 |
|  | R | 122 | 2 | 2 | 0 | 0 | 1 | 0 | 1 | 0 | 1 | 1 | 0 |
| VII | L | 497 | 4 | 5 | 3 | 4 | 5 | 6 | 3 | 4 | 1 | 4 | 6 |
|  | R | 594 | 2 | 4 | 4 | 2 | 5 | 3 | 3 | 4 | 4 | 3 | 1 |
| VIII | L | 106 | 1 | 1 | 1 | 1 | 1 | 1 | 1 | 2 | 1 | 1 | 1 |
|  | R | 457 | 1 | 2 | 1 | 3 | 6 | 3 | 3 | 5 | 3 | 4 | 0 |
| IX | L | 356 | 3 | 2 | 3 | 2 | 2 | 1 | 2 | 1 | 3 | 2 | 1 |
|  | R | 84 | 0 | 1 | 0 | 0 | 0 | 0 | 0 | 0 | 0 | 0 | 1 |
| X | L | 436 | 3 | 2 | 3 | 2 | 2 | 4 | 4 | 4 | 4 | 1 | 0 |
|  | R | 309 | 2 | 2 | 1 | 3 | 1 | 3 | 4 | 2 | 1 | 4 | 3 |
| XI | L | 440 | 4 | 2 | 2 | 2 | 2 | 1 | 1 | 3 | 3 | 2 | 4 |
|  | R | 227 | 2 | 1 | 2 | 0 | 2 | 3 | 0 | 1 | 3 | 1 | 3 |
| XII | L | 151 | 1 | 1 | 1 | 1 | 0 | 0 | 1 | 1 | 1 | 1 | 1 |
|  | R | 927 | 4 | 6 | 8 | 3 | 6 | 4 | 5 | 4 | 9 | 6 | 4 |
| XIII | L | 268 | 1 | 2 | 3 | 1 | 2 | 2 | 1 | 4 | 1 | 2 | 3 |
|  | R | 656 | 6 | 5 | 4 | 6 | 4 | 5 | 4 | 3 | 4 | 6 | 5 |
| XIV | L | 629 | 3 | 4 | 5 | 5 | 4 | 1 | 5 | 4 | 6 | 4 | 3 |
|  | R | 156 | 2 | 1 | 0 | 0 | 3 | 2 | 1 | 0 | 1 | 2 | 2 |
| XV | L | 327 | 2 | 1 | 2 | 1 | 3 | 3 | 2 | 2 | 2 | 2 | 2 |
|  | R | 765 | 5 | 7 | 4 | 6 | 3 | 5 | 6 | 4 | 6 | 3 | 6 |
| XVI | L | 556 | 1 | 2 | 3 | 3 | 4 | 6 | 2* | 4 | 2 | 2 | 4 |
|  | R | 392 | 2 | 1 | 3 | 4 | 4 | 1 | 3 | 4 | 5 | 2 | 4 |
| SUM |  |  | 70 | 76 | 73 | 71 | 84 | 79 | 81 | 80 | 83 | 80 | 81 |

*ChrXVI Left Arm of hhhs2 show a chromosome instability.

**Supplementary Table 7** Sequence reads from Spo11 bound or MNase-resistant oligos

|  | **Item** | **Diploid** | **Tetraploid** |
| --- | --- | --- | --- |
| Spo11 ChIP-seq | total reads | 1909271 | 1266768 |
|  | Spo11 bound reads at DSB sites | 365198 | 278176 |
|  | Spo11 bound reads at control sites | 135874 | 84528 |
|  | Percent of Spo11 bound reads at DSB sites | 0.191276 | 0.219595 |
|  | Percent of Spo11 bound reads at control sites | 0.071165 | 0.066727 |
| MNase-seq | total reads | 3165089 | 3221318 |
|  | MNase-resistant reads at DSB sites | 122282 | 110412 |
|  | MNase-resistant reads at control sites | 158404 | 163549 |
|  | Percent of MNase-resistant reads at DSB sites | 0.038635 | 0.034275 |
|  | Percent of MNase-resistant reads at control sites | 0.050047 | 0.050771 |

**Supplementary Figure 2**

Diploid hs1（S288C/SK1）

Haploid gametes （**S288C**, **SK1**）


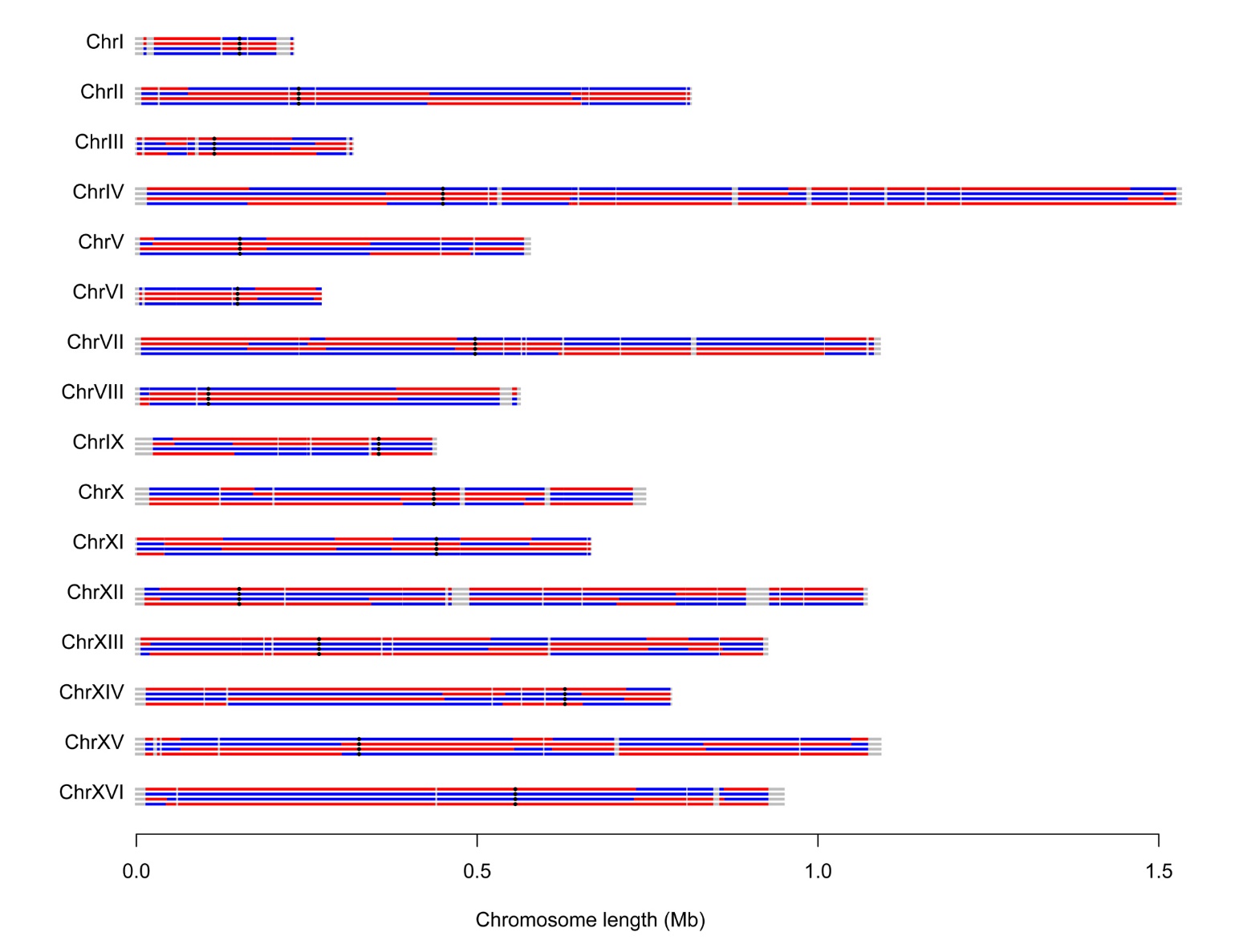


Diploid hs2（S288C/SK1）

Haploid gametes （**S288C**, **SK1**）


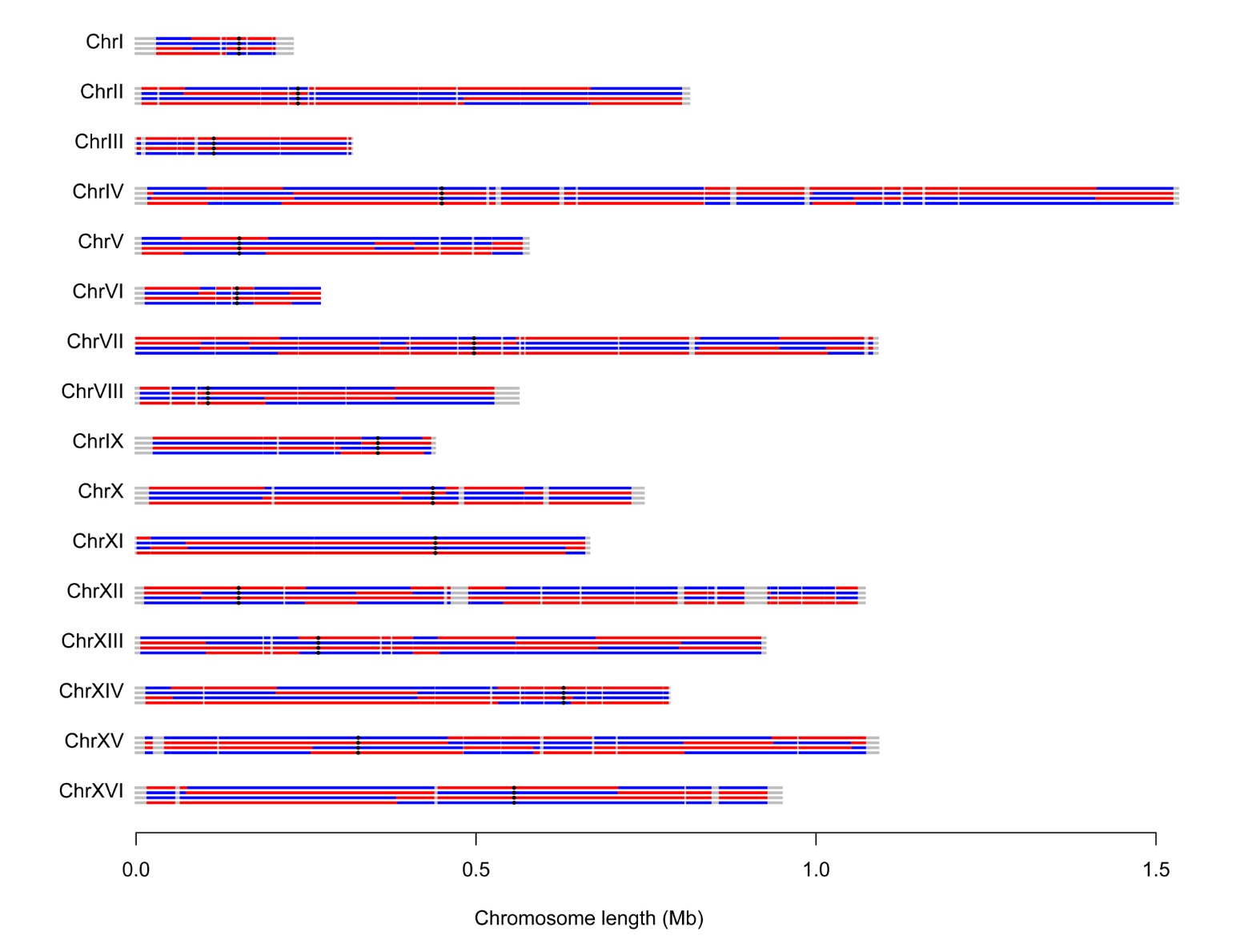


Diploid hs3（S288C/SK1）

Haploid gametes （**S288C**, **SK1**）


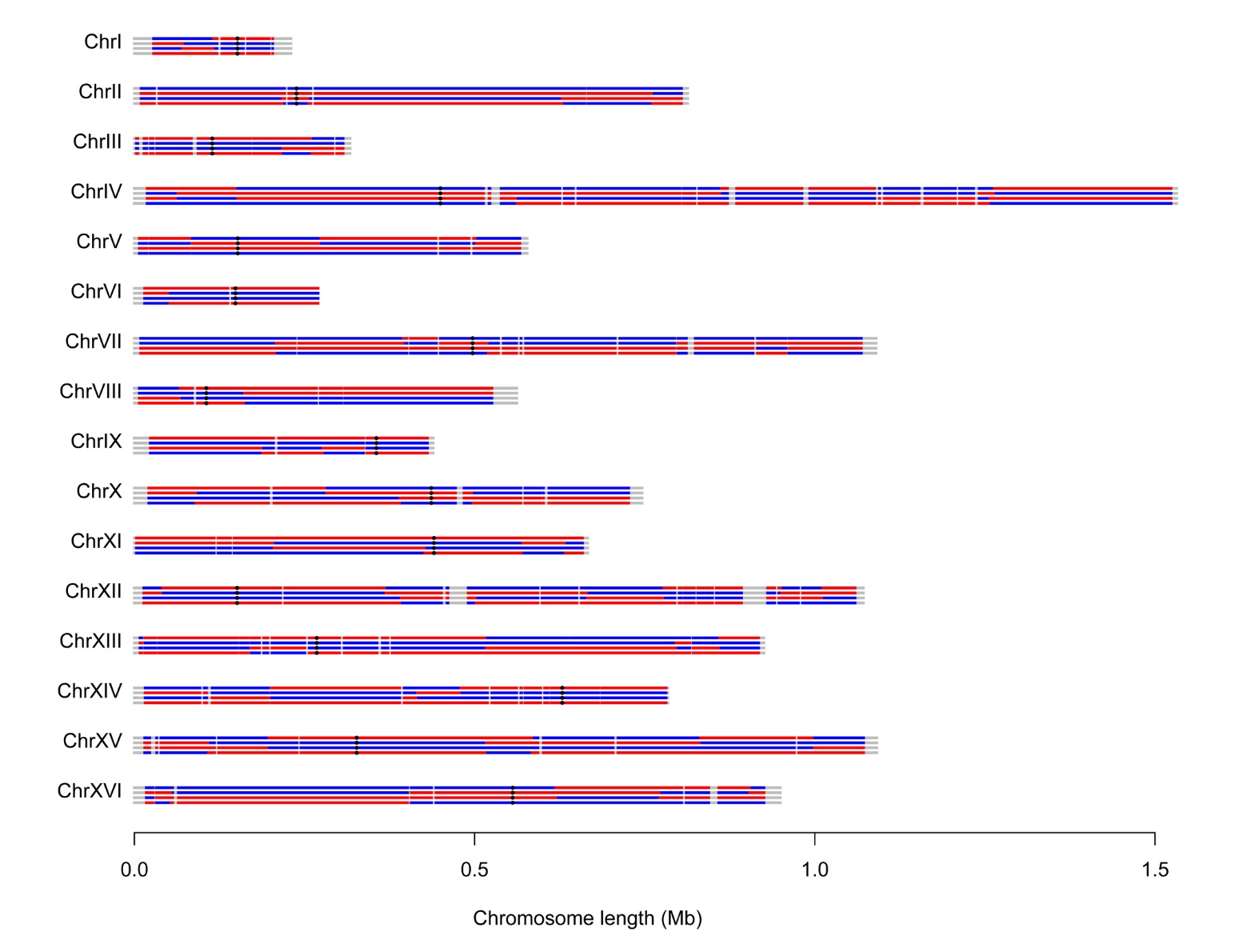


Diploid hs4（S288C/SK1）

Haploid gametes （**S288C**, **SK1**）


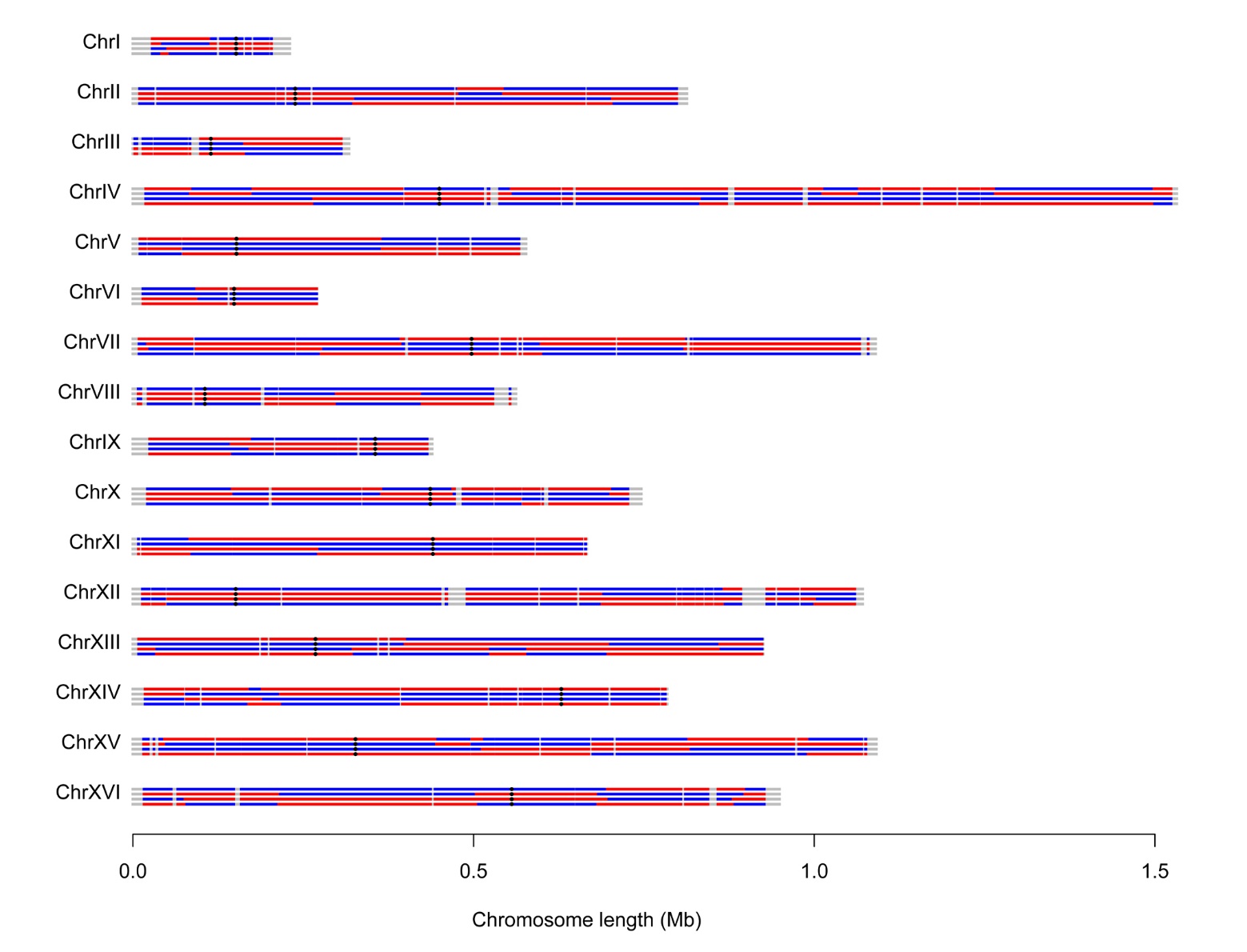


Diploid hs5（S288C/SK1）

Haploid gametes （**S288C**, **SK1**）


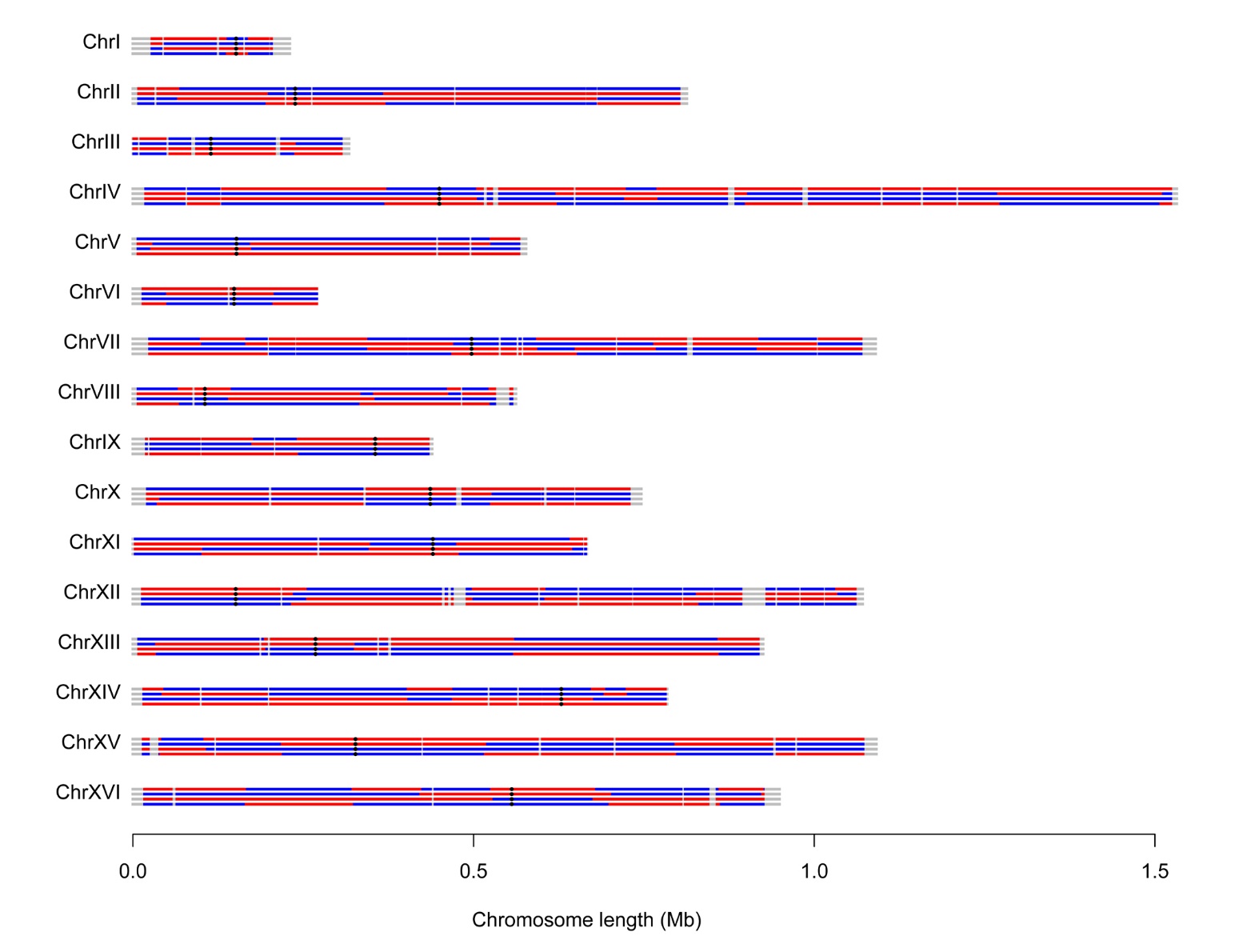


Tetraploid hhhs1（S288C/S288C/S288C/SK1）

Diploid gametes （**S288C/S288C**, **SK1/SK1, S288C/SK1**）


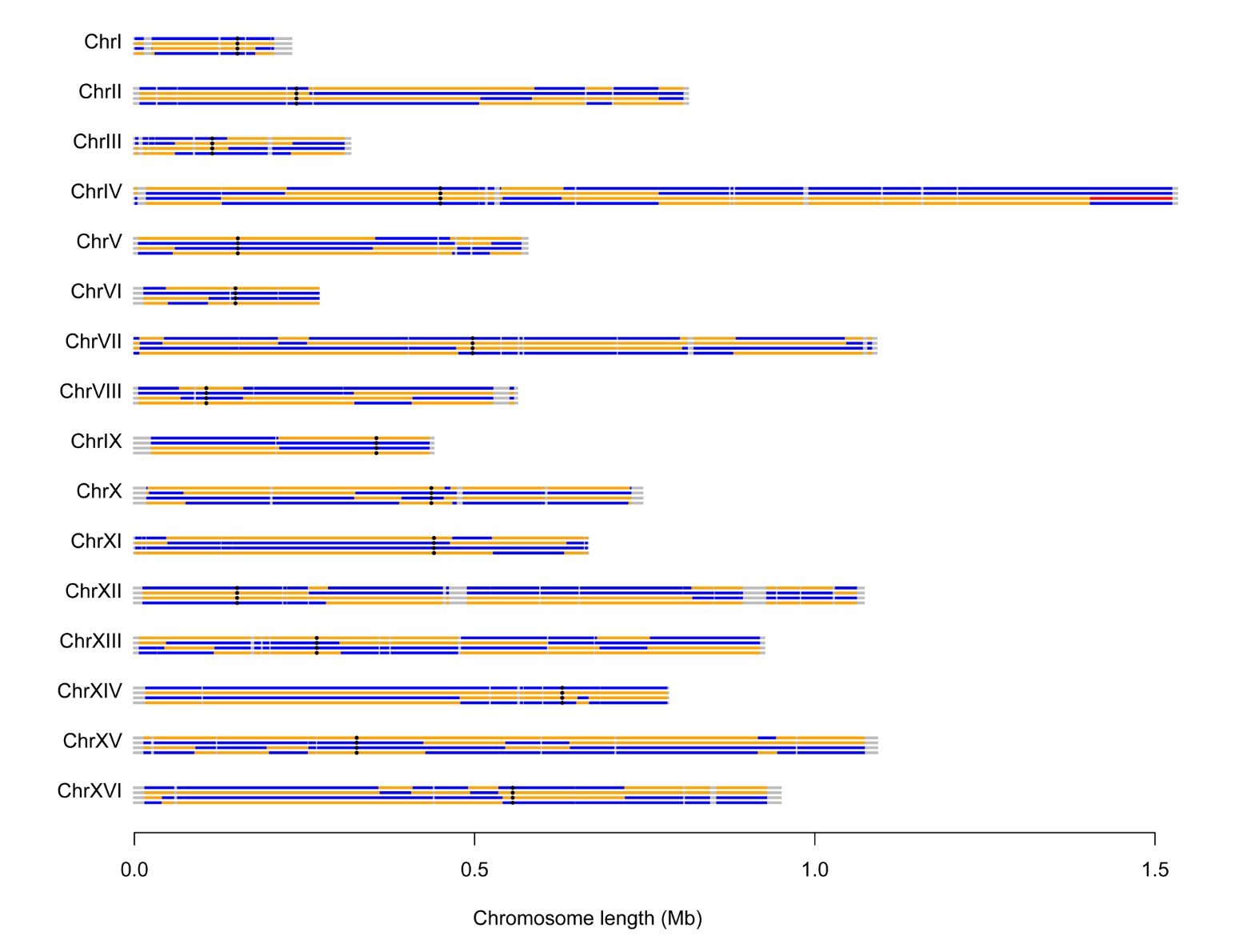


Tetraploid hhhs2（S288C/S288C/S288C/SK1）

Diploid gametes （**S288C/S288C**, **SK1/SK1, S288C/SK1**）


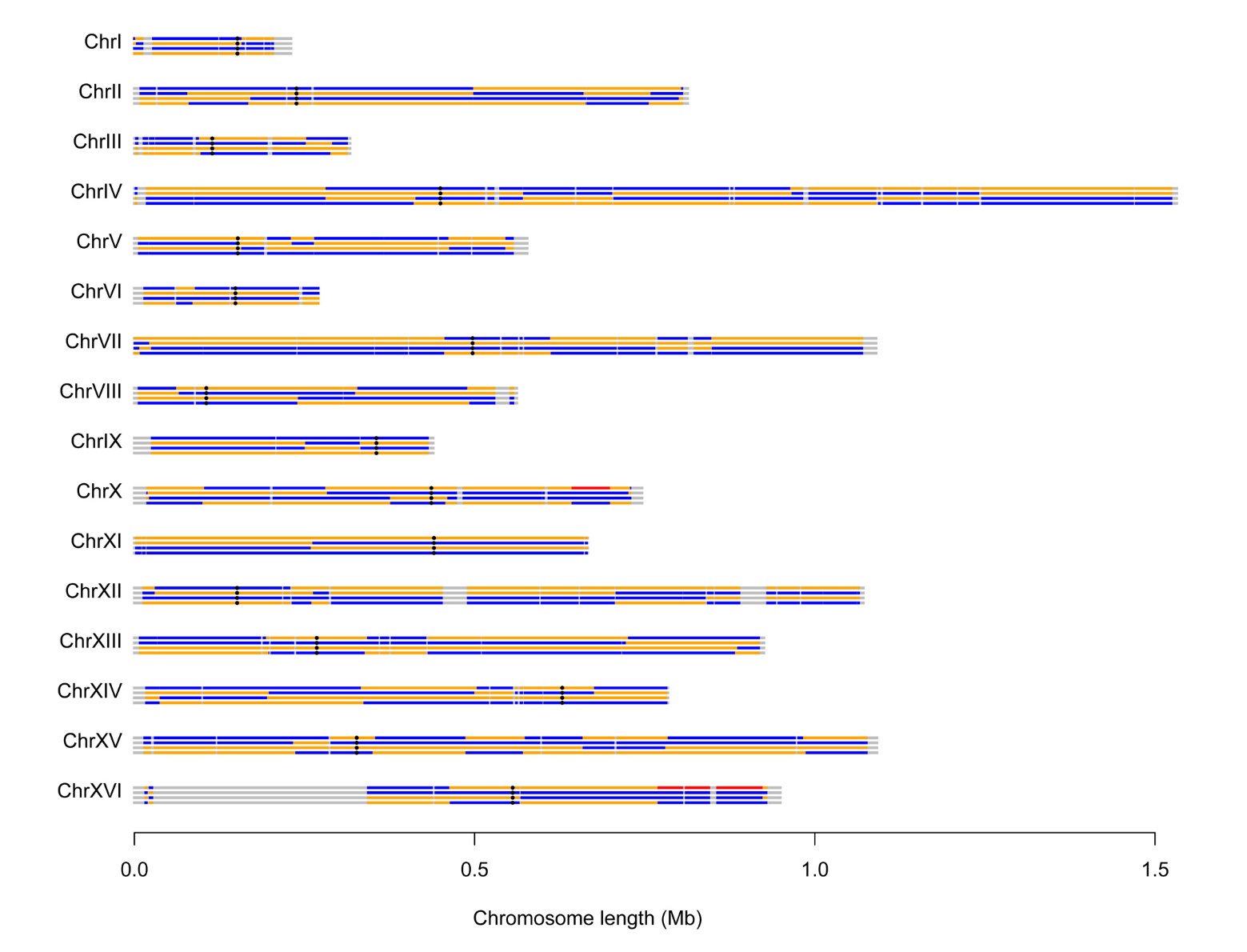


Tetraploid hhhs3（S288C/S288C/S288C/SK1）

Diploid gametes （**S288C/S288C**, **SK1/SK1, S288C/SK1**）


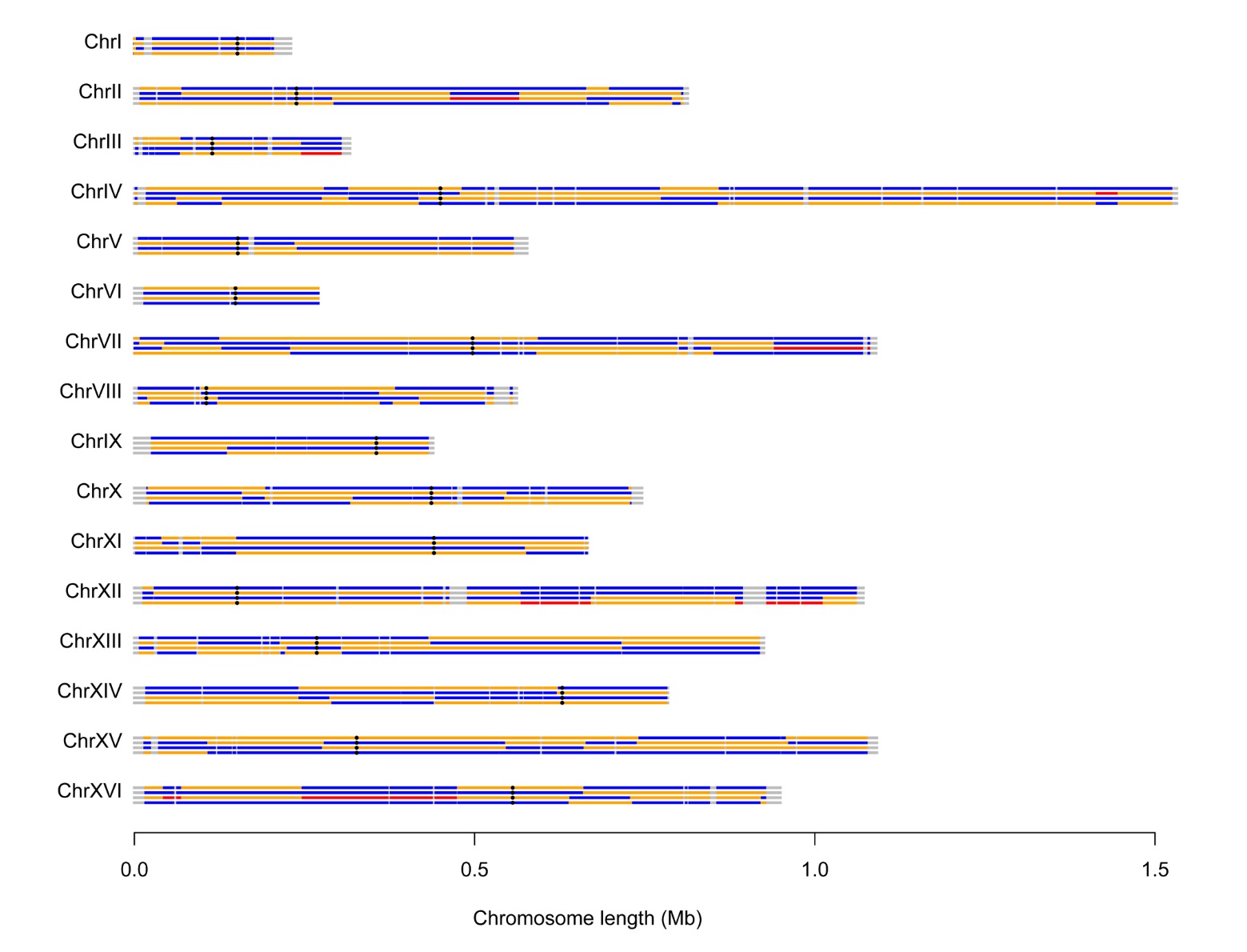


Tetraploid hsss1（S288C/SK1/SK1/SK1）

Diploid gametes （**S288C/S288C**, **SK1/SK1, S288C/SK1**）


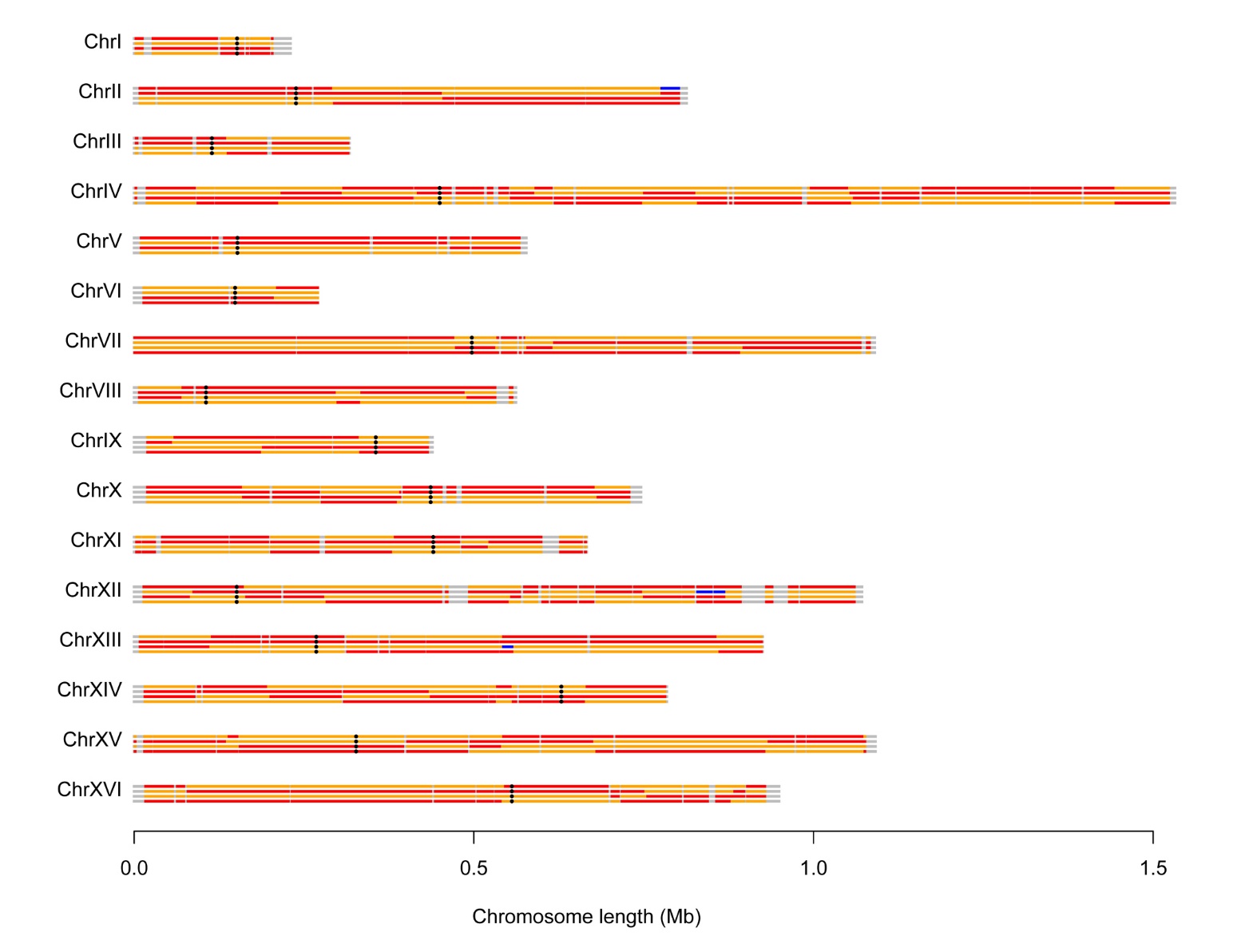


Tetraploid hsss2（S288C/SK1/SK1/SK1）

Diploid gametes （**S288C/S288C**, **SK1/SK1, S288C/SK1**）


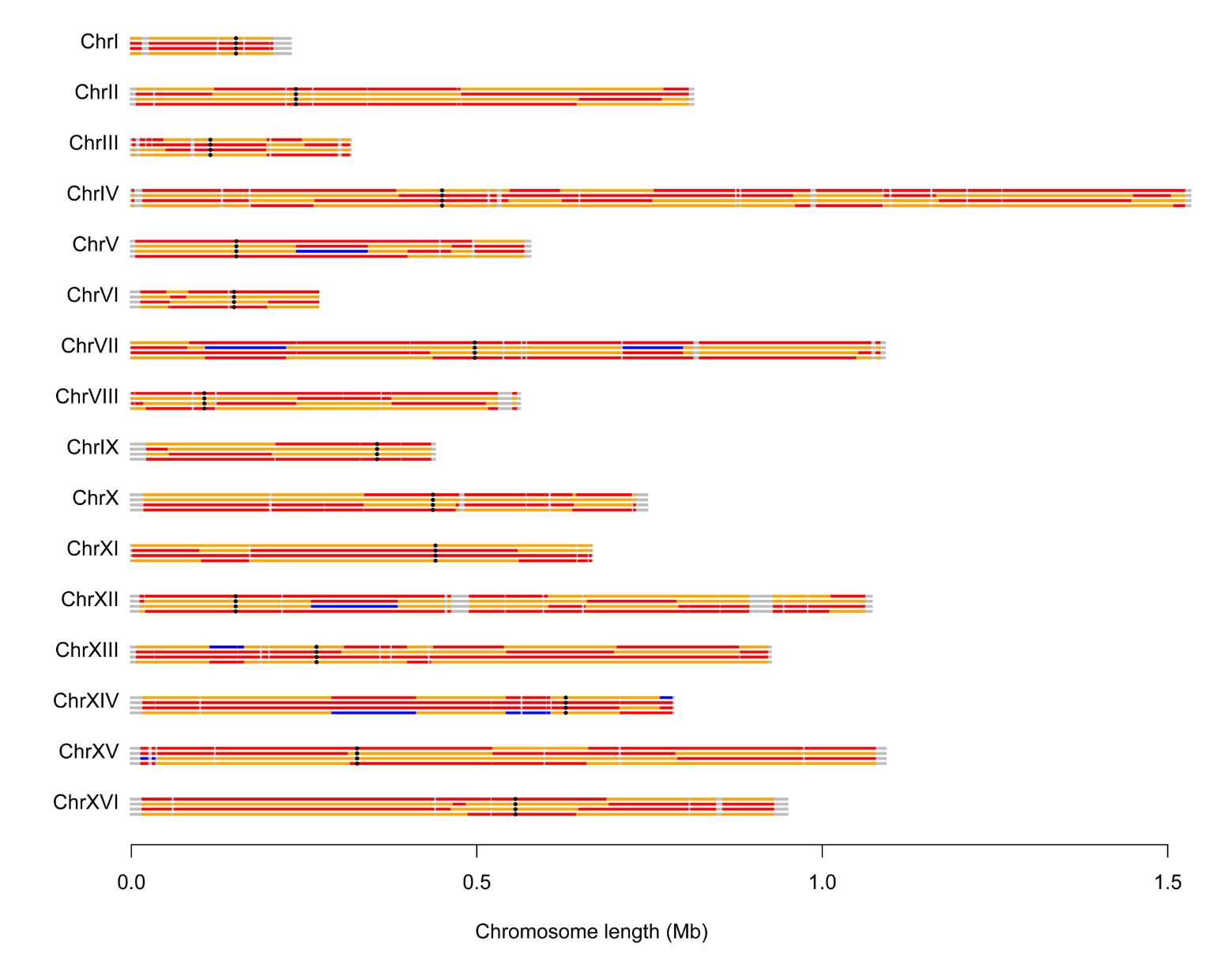


Tetraploid hsss3（S288C/SK1/SK1/SK1）

Diploid gametes （**S288C/S288C**, **SK1/SK1, S288C/SK1**）


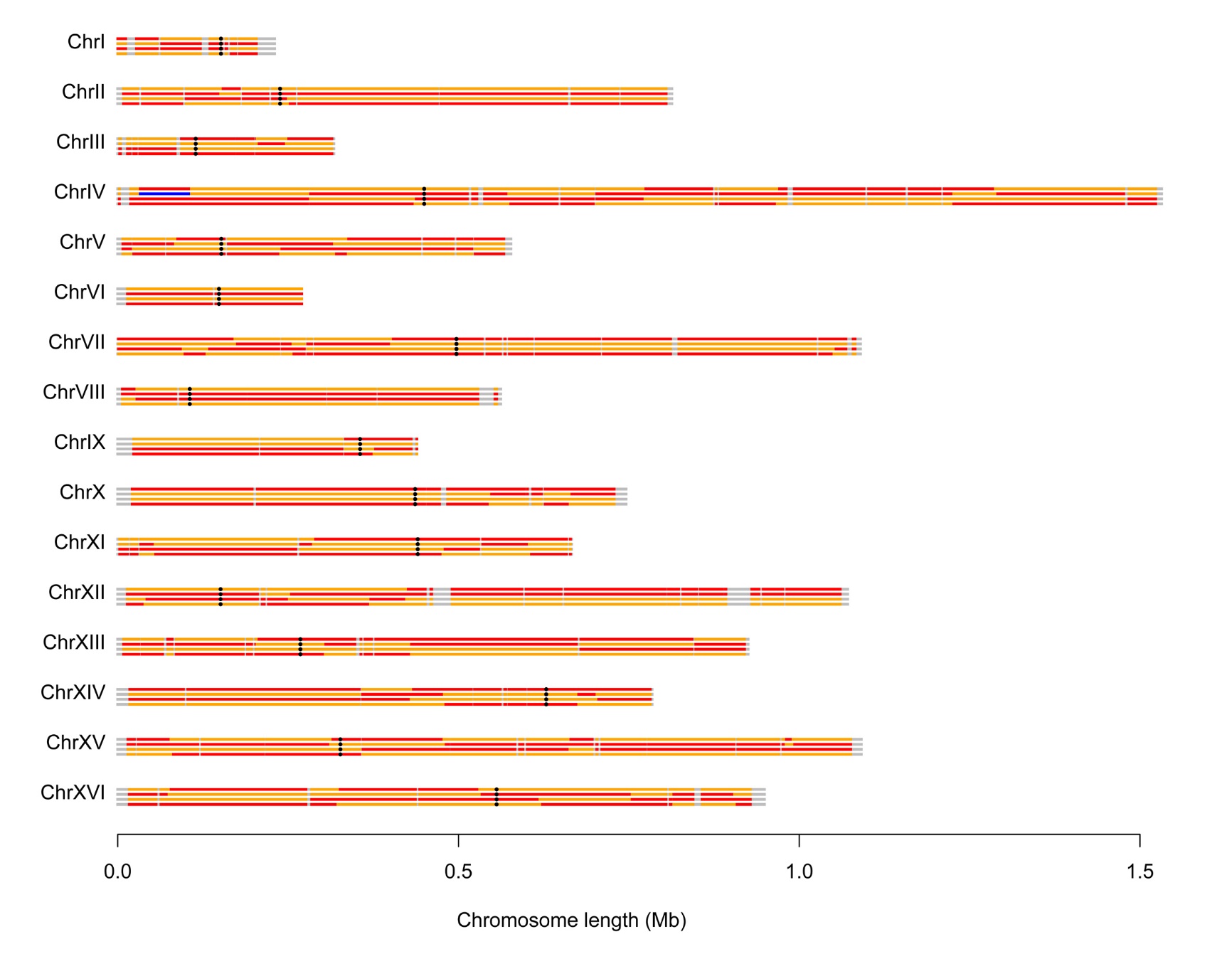


**Supplementary Materials and Methods**

**Plasmid construction**

All plasmids were propagated in *E. coli* strain *TOP10* in the LB medium (0.5% Yeast extract, 1% Polypeptone, 1% NaCl) added with 100 μg/ ml ampicillin. *pTetra* was constructed from modifying the plasmid *YCP50* ^[1]^. The *P_HO_-natMX4-T_HO_* cassette was first amplified through a polymerase chain reaction (PCR) and then cloned into *YCP50* at the *Ecor*I and *BamH*I sites. *hphMX4* from *PAG32* ^[2]^ was then used to replace the selection marker *URA3* at the *Sal*I and *Nsi*I sites. Finally, PCR amplified *P_GAL10_-HO-T_HO_* cassette was ligated into the vector at the *Xho*I site. *pGFP* and *pRFP* were both constructed from *pUG6* ^[3]^. The *GFP* and *RFP* cassettes were engineered using overlap extension PCR. The promoter DNA of 1170 bp (upstream of the start codon of *TEF2*), *GFP* ORF from *pFA6a-GFPS65T-HIS3MX6* ^[4]^ and *AOX1* terminator from *pAGZA*(Invitrogen) were first overlapped by PCR to build the *pGFP* cassette which was then fused into *pUG6* at *Sal*I site. Finally, the anti-G418 *kanMX* in the plasmid was replaced by *hphMX4* at *Bgl*II and *Sac*I sites. Similarly, 657bp upstream of *TDH3,* *REDSTAR* ORF from *pYM38* ^[5]^ and *CYC1* terminator from *pSH47* ^[3]^ were overlapped by PCR to create the *pRFP* *cassette* which was in turn introduced into *pUG6* at the *Sal*I site. *kanMX* was finally replaced by *natMX4* in the newly constructed plasmid ^[2]^.

**Duplicating a diploid yeast genome and confirmation of ploidy level**

To create autotetraploid yeast, we firstly switched wild type *MATa/α* diploid strain into the diploid strain with a mating type of *MATa/a* or *MATα/α*^[6]^. Details were as follows: firstly, plasmid *pTetra* was transformed into the *MATa/α* diploid. After a brief exposure to YPGal, the diploid cells were then streaked out onto YPD plate added with clonNat. When single colonies grew, their mating types were checked and verified after mating with a standard strain, together with the PCR assay, using the primers “AGTCACATCAAGATCGTTTATGG, GCACGGAATATGGGACTACTTCG and ACTCCACTTCAAGTAAGAGTTTG” ^[7]^ . Autotetraploid trains were constructed by fusing diploids with *MATa/a* and *MATα/α* mating types. DNA in the nuclei of these artificially constructed antotetraploid strains was stained by propidium iodide and the ploidy levels were checked using a flow cytometry.

**Assay of meiotic recombination frequency using spore-autonomous fluorescent marker system**

The lithium acetate method ^[8]^ was implemented to transform the two fluorescent cassettes, GFP and RFP, into the same chromosome at pre-designed locations in the haploid strain s288c with the matting type *MATa* (primers were listed in Supplementary table 5)*.* The genetically transformed haploid was used to construct the diploid or autotetraploid yeast strains carrying fluorescent markers in single copy and in *cis*. Specifically, the diploid strain was made by fusing the haploid carrier of the fluorescent marker cassette with the wild-type haploid strain SK1 with an opposite mating type*.* The fluorescent maker carrier diploid was then switched mating type and fused with a wild-type diploid (s288c/SK1 hybrid) to construct the fluorescent marker cassette carrying autotetraploid as described above. Sporulation assay was performed as described ^[9]^. The diploids and autotetraploid cells carrying the fluorescent marker cassette were streaked out to create single colonies on the YPD plate. After 2 days of colony development, 3 large and healthy colonies were patched on a new YPD plate and grew for 13.5 hours, then transferred to the SPM plate (1% KAC) and incubated at 30 °C for 3-4 days. Autonomous fluorescent tetrads were visualized and scored on a fluorescent microscopy (Olympus, IX71) with 100x amplification. Microscope images were acquired and analyzed using the software DP2-BSW.

**Crossover interference assay**

The lithium acetate method was also implemented to transform the three anti-biotic genes *hphMX4* (anti-hygromycin B), *natMX4* (anti-nourseothricin), *kanMX4* (anti-G418), into a given chromosome at pre-designed locations in the haploid strain s288c with *MATa.* The transformation of the anti-biotic genes was repeatedly and respectively done in yeast chromosomes III, VI and VIII. The genetically modified haploid strain was then used to construct diploid and autotetraploid strains in the same way as described above. Right after completion of meiosis, tetrads generated from the diploid and autotetraploid strains were dissected by use of a micromanipulator (Singer, MSM300). Single-colony cultures were patched on a YPD plate added with hygromycin B, a YPD plate added with nourseothricin, a YPD plate added with G418 and a standard YPD plate. Genotype of each spore was confirmed from whether it could grow on a plate added with each configuration of the three anti-biotics.

**Tetrad genomic DNA sequencing**

Diploid or autotetraploid strains sporulated in the way described above, and the tetrads were dissected by use of a micromanipulator (Singer, MSM300). Genomic DNA was then extracted from single-colony cultures of the tetrads and sheared into fragments with an average length of 200 bp using the Covaris S220 (Duty Factor=10%, Intensity Peak Incident Power = 140W, Cycles per Burst = 200，Processing Time = 180 seconds，Volume = 130μl in microtubes). The DNA fragments were then purified by use of the QIAGEN minelute gel extraction kit. Sequencing library was prepared using the NEBNext Ultra DNA Library Prep Kit designed for Illumina and whole genome sequencing was performed using an Illumina Hiseq-2000 sequencer with a design to generate 2 x 100 bp paired end reads and designed coverage of 10X for haploid gametes, and 15x for diploid gametes.

**Meiosis synchronization**

The protocol for meiosis synchronization was implemented as previously described ^[10]^ with some modifications in the present study. Cells were patched onto YPG plates from a -80°C frozen stock and grown at 30°C overnight. The cell culture was streaked out to single colonies on YPD plates and grown for other two days. A healthy single colony was then transferred to 5 ml YPD liquid medium and grown for 24 hours and then 200 (or 400μl) diploid (or autotetraploid) cell culture was added into 100ml SPS medium. After incubated for other 18 hours at 30°C, cells would be grown to reach a concentration of OD600=3.5~4.0. SPS culture was then pelleted, washed with SPM medium and incubated in 200ml SPM medium with vigorous shaking. Meiotic division of test cells was effectively monitored by a fluorescence microscope after the cells were stained with 0.2 μg/mL DAPI.

**ChIP-seq assay**

The protocol for ChIP was implemented as previously described with modifications ^[11]^. Specifically, 50 ml of diploid and autotetraploid cultures synchronized at meiosis were harvested at 5 hours after being transferred into sporulation, and washed three times with icy-cold 1× TBS (20 mM Tris-Cl at pH 7.5, 150 mM NaCl). The cells were pelleted and re-suspended in 500 μl of lysis buffer (50 mM HEPES-KOH at pH 7.5, 140 mM NaCl, 1 mM EDTA, 1% Triton X-100, 0.1% Na-deoxycholate, 1 tablet of complete inhibitor cocktail (Roche) every 10 mL solution) and lysed with acid-washed glass beads for 15 minutes in a vortex at full output. After the cell debris was removed via centrifuging at 12000 rpm for 5 minutes at 4°C, chromatin in the supernatant was sheared to fragments with lengths varying between 200 bp to 500 bp using Covaris S220 (Duty Factor=5%, Intensity Peak Incident Power = 105W, Cycles per Burst = 200，Processing Time = 20 minutes, Volume=1ml in TC16 tubes). Immuno-precipitation (IP) was achieved in 20 μl magnetic dynabeads protein G (Invitrogen) which was added with 2.5 mg (1000 μl) extract after being incubated with the monoclonal mouse anti-Myc (Sigma, 9E10) antibody for 2 hours at room temperature. Precipitates were washed in order with lysis buffer, lysis buffer plus 360 mM NaCl, washing buffer (10 mM Tris-Cl at pH 8.0, 250 mM LiCl, 0.5% NP-40, 0.5% Na-deoxycholate, 1 mM EDTA), and 1× TE at pH 7.5 with aid of the magnetic device supplied by Dynal. The precipitated DNA was eluted by heating in TES for 30 minutes at 65°C, then digested with proteinase K (Merck) for 1 hour at 37°C. The DNA was finally recovered using the PCR Purification Kit (QIAGEN). Spo11-oligos’ 5’ ends could be blocked by a covalent linkage with residual amino acids of Spo11 protein and thus affecting the feasibility of sequencing library construction ^[12]^. To overcome this problem we performed another round of DNA shearing by use of Covaris S220 in a 130 μl microtube with setting Duty Factor = 10%, Intensity Peak Incident Power = 175W, Cycles per Burst = 200，and Processing Time equals 430 seconds. Sheared DNA was purified by use of the PCR Purification Kit (QIAGEN) and prepared into sequencing library using the NEBNext ChIP-Seq Library Prep Master Mix Set for Illumina (NEB). The library was sequenced on an Illumina Miseq sequencer to generate more than 1.5 million reads 1 x 50 single end reads.

**Mononucleosomal DNA sequencing**

The protocol for mononucleosomal DNA sequencing was implemented as previously described with modifications ^[13]^. Specifically, 50 ml cultures of diploid or autotetraploid cells that were synchronized at meiosis were harvested, and were immediately crosslinked in 1.4 ml formaldehyde (37%) and incubated for 15 minutes at room temperature. The crosslink was terminated in presence of 2.7 ml Glycine (2.5M), cells were pelleted and re-suspended in 0.5 ml Buffer Z (0.5 M sorbitol, 50 mM Tris-HCl, pH 7.4, 10 mM β-mercaptoethanol) containing 15 U lyticase (Sigma) and incubated at 30°C for 30 minutes. Spheroplasted cells were pelleted, treated with 800U micrococcal nuclease (NEB) in NP buffer (1 M sorbitol, 50 mM NaCl, 10 mM Tris-HCl, pH 7.4, 5 mM MgCl_2_, 1 mM CaCl_2_, 1 mM β-mercaptoethanol, 0.075% NP-40) and incubated at 37°C for 20 minutes. EDTA and RNase A were then added to the spheroplasted cells and incubated at room temperature for other 20 minutes, 50μl 10% SDS and 20μl proteinase K (20mg/ml, Merck) were added in, and finally incubated at 65°C for 18 hours. DNA was extracted by use of phenol-chloroform-isoamyl alcohol (25:24:1), and precipitated with ethanol. Mononucleosome sized DNA was then purified by size fractionation on 2% agarose gels and purified using QIAGEN minelute gel extraction kit. The purified DNA was prepared into sequencing library by use of the NEBNext Ultra DNA Library Prep Kit, and was then sequenced by an Illumina Mi-seq sequencer for generating more than 1.5 million reads 1 x 50 single end reads.

**Southern blotting based DSB test**

50 ml of diploid and autotetraploid cultures synchronized at meiosis were harvested at 5 hours after being transferred to sporulation condition. Genomic DNA of test samples was isolated and digested with the restriction enzyme AseI (YCR047C site, fermentas) or BglII (YFR025C-A, B site, fermentas) overnight at 37°C. The digested DNA was purified by quick PCR Purification Kit (QIAGEN), and then quantified using Qubit (Invitrogen). 2 μg of the purified DNA was loaded and separated in 0.8% agarose gels through electrophoresis at 60 V for 2 hours, and then transferred to the positively charged nylon membrane using capillary transfer method ^[14]^. Southern blot hybridization was performed using DIG-11-dUTP labeled DNA fragment as probe. Primer sequences for amplification of probes were CTTTCACACCTGAAGAGCAAG, GTGCAATGAGTACAATGGAGAC (YCR047C site) or GACAGATTCCCAT GAAGGTG, GCACCCTGGGTG TTAAGG (YFR025C-A and B sites). Chemiluminescent detection of the DIG-labeled hybrids was performed using High Prime DNA Labeling and Detection Starter Kit II (Roche) and quantified using the Quantity One software (Bio-Rad). DSB frequency was calculated as the ratio (percentage) of gray value for DSB fragments over total gray value in the lane ^[15]^.

**Random Spore Analysis**

Genetically modified diploid or autotetraploid yeast cells carrying *his2-A* and *his2-X* alleles were induced to enter meiosis synchronization in the way described above. The cell samples were collected and pelleted at 24 hours after synchronization, and then incubated in 50% diethyl ether to kill the vegetative cells that had undergone an incomplete meiosis ^[16]^. Remaining tetrad asci were digested with lyticase, then diluted in 0.1% Tween-20 and, in turn, sonicated. Properly diluted ascus samples were plated on YPD plate to measure total viable cells and, in parallel on the synthetic complete medium (SC) in lack of histidine plate (SC-his) to measure His+ prototroph formation. Grown colonies were counted at 2 or 3 days after the YPD and SC-his plates cultivated at 30°C.

**Reference**

1. Rose, M. D.,Novick, P.,Thomas, J. H., etc., A Saccharomyces cerevisiae genomic plasmid bank based on a centromere-containing shuttle vector. Gene. 1987; 60 : 237-43.
2. Goldstein, A. L.,McCusker, J. H., Three new dominant drug resistance cassettes for gene disruption in Saccharomyces cerevisiae. Yeast. 1999; 15 (14): 1541-53.
3. Guldener, U.,Heck, S.,Fiedler, T., etc., A new efficient gene disruption cassette for repeated use in budding yeast. Nucleic Acids Research. 1996; 24 (13): 2519-2524.
4. Wach, A.,Brachat, A.,Alberti-Segui, C., etc., Heterologous HIS3 marker and GFP reporter modules for PCR-targeting in *Saccharomyces cerevisiae*. Yeast. 1997; 13 (11): 1065-75.
5. Janke, C.,Magiera, M. M.,Rathfelder, N., etc., A versatile toolbox for PCR-based tagging of yeast genes: new fluorescent proteins, more markers and promoter substitution cassettes. Yeast. 2004; 21 (11): 947-62.
6. Fang O, Hu X, Wang L, et al. Amn1 governs post-mitotic cell separation in Saccharomyces cerevisiae. PLoS genetics, 2018, 14(10): e1007691.
7. Huxley, C.,Green, E. D.,Dunham, I., Rapid assessment of S. cerevisiae mating type by PCR. Trends in Genetics. 1990; 6 (8): 236.
8. Gietz, R. D.,Woods, R. A., Transformation of yeast by lithium acetate/single-stranded carrier DNA/polyethylene glycol method. Methods Enzymol. 2002; 350: 87-96.
9. Li, J.,Wang, L.,Wu, X., etc., Polygenic molecular architecture underlying non-sexual cell aggregation in budding yeast. DNA Res. 2013; 20 (1): 55-66.
10. Kim, K. P.,Weiner, B. M.,Zhang, L., etc., Sister cohesion and structural axis components mediate homolog bias of meiotic recombination. Cell. 2010; 143 (6): 924-37.
11. Prieler, S.,Penkner, A.,Borde, V., etc., The control of Spo11's interaction with meiotic recombination hotspots. Genes Dev. 2005; 19 (2): 255-69.
12. Keeney, S.,Giroux, C. N.,Kleckner, N., Meiosis-specific DNA double-strand breaks are catalyzed by Spo11, a member of a widely conserved protein family. Cell. 1997; 88 (3): 375-384.
13. Kaplan, N.,Moore, I. K.,Fondufe-Mittendorf, Y., etc., The DNA-encoded nucleosome organization of a eukaryotic genome. Nature. 2009; 458 : 362-6.
14. Chong, L., Molecular cloning - A laboratory manual, 3rd edition. Science. 2001; 292: 446-446.
15. Murakami, H.,Borde, V.,Nicolas, A., etc., Gel electrophoresis assays for analyzing DNA double-strand breaks in Saccharomyces cerevisiae at various spatial resolutions. Methods Mol Biol. 2009; 557: 117-42.
16. Dawes, I. W.,Hardie, I. D., Selective killing of vegetative cells in sporulated yeast cultures by exposure to diethyl ether. Mol Gen Genet. 1974; 131 (4): 281-9.
